# Supplementary material for: Glycosaminoglycans from the Starfish Lethasterias fusca: Structures and Influence on Hematopoiesis
Source: Mar Drugs. 2023 Mar 24;21(4):205. doi: 10.3390/md21040205 (PMC10146216; doi:10.3390/md21040205)
Supplement: Supplementary file 1 [file marinedrugs-21-00205-s001.zip › marinedrugs-2282082-supplementary.pdf]

Supplementary data

Glycosaminoglycans from the starfish *Lethasterias fusca*: structures and influence on hematopoiesis

Maria I. Bilan, Natalia Yu. Anisimova, Alexandra I. Tokatly, Sofya P. Nikogosova, Dmitriy Z. Vinnitskiy, Nadezhda E. Ustyuzhanina, Andrey S. Dmitrenok, Evgenia A. Tsvetkova, Mikhail V. Kiselevskiy, Nikolay E. Nifantiev, Anatolii I. Usov

3.5. Model monosaccharide synthesis

3.5.1. General procedures

3.5.1.1. General procedure A: tert-butyldimethylsilyl ether cleavage

A1, for unsulfated compounds: The TBS-protected monosaccharide (0.05 mmol) was dissolved in THF (1mL), a solution of TBAF monohydrate (3eq.) and AcOH (1 eq.) in 0.3 mL THF was added, reaction mixture was stirred for 1 h at room temperature, diluted with EtOAc and washed with H_2O. The organic layer was concentrated and purified by chromatography (silica gel, eluent hexane:EtOAc).

A2, for sulfated compounds: The TBS-protected monosaccharide (0.03 mmol) was dissolved in THF (1mL), a solution of TBAF monohydrate (12 eq.) in 0.3 mL THF was added, reaction mixture was stirred at 50 °C overnight, evaporated, purified via column chromatography (silica gel, eluent DCM:MeOH) to yield the deprotected product as a TBA salt.

3.5.1.2. General procedure B: 2-methyl-naphthyl ether cleavage

The starting compound (0.05 mmol) was dissolved in DCM (1.8 mL), then H_2O (0.2 mL) and DDQ (1.2 eq) were added and the mixture was stirred vigorously for 3 h. Upon completion the mixture was diluted with EtOAc and washed with 1M $Na_2S_2O_3$ and H_2O, the organic layer was dried over Na_2SO_4 and concentrated *in vacuo*. The residue was purified by chromatography (silica gel, eluent hexane/EtOAc).

3.5.1.3. General procedure C: selective benzoyl deprotection

The starting monosaccharide (0.06 mmol) was dissolved in MeOH (1.2 mL) and K_2CO_3 (2 eq.) was added under Ar protection. The mixture was stirred for 1 h, when TLC showed full consumption of the starting material it was quenched with 1M AcOH and evaporated, then purified by column chromatography (silica gel, eluent: hexane/EtOAc).

3.5.1.4. General procedure D: Sulfation

To a solution of the deprotected monosaccharide (0.05 mmol) in DMF (1 mL) Py·SO_3 complex (4 eq.×-OH) was added. The reaction mixture was kept at rt for 30 minutes, then quenched with excess of $NaHCO_3$ (5 eq×-OH) and MeOH (0.1 mL), then stirred for 3h and evaporated to dryness. The product was purified either by chromatography (silica gel: DCM/MeOH) or by gel-chromatography on a Sephadex G-15 column to give the desired product.

3.5.1.5. General procedure E: Saponification

E1, methyl uronate cleavage: To a solution of starting compound (0.05 mmol) in H_2O (0.5 mL) 2M solution of LiOH in H_2O (0.2 mL) was added at 0 °C. The reaction mixture was kept at 0 °C until TLC showed formation of the final product, then quenched with 0.5 M AcOH and evaporated to dryness.

E2, benzoyl protection cleavage: After addition of LiOH and cleavage of the methyl uronate ester as per E1, 1M NaOH (0.2 mL) was added at room temperature and the mixture was stirred for 2 hours, then quenched with AcOH and evaporated.

3.5.1.6. General procedure F: Hydrogenolysis

Starting compound (0.05 mmol) was dissolved in THF/H_2O (5:1, 600μL), 20% $Pd(OH)_2/C$ (30 mg) was added; the mixture was stirred overnight under H_2 at room temperature, then filtered through a nylon membrane syringe filter (0.45 μm). The filtrate was concentrated *in vacuo* and purified by gel permeation chromatography on a Toyopearl HW-40S column.to give the final product.

3.5.2. (2-methyl-5-tert-butylphenyl) 3-O-(2-methylnaphthyl)-1-thio-β-D-glucopyranoside ***(2)***

Thioglycoside **1** (Martin, Weishaupt, & Seeberger, 2011) (6.1 g, 12 mmol) was dissolved in MeOH (12 mL) and 1 M solution of MeONa (3 mL) was added. The mixture was stirred at 40 °C for 15 minutes and neutralized with ion exchange resin IR-120 in H^+ form when TLC (EtOAc) showed the single final product remaining. The resin was filtered off and the filtrate was concentrated *in vacuo*. The crude residue was dissolved in CH_3CN (14 mL), CSA was added (140 mg) followed by $HC(OMe)_3$ (2.2 mL, 13 mmol) and *p*-anisaldehyde (1.6 mL, 20 mmol). After 20 min TLC showed full consumption of the staring material, the reaction mixture was diluted with EtOAc (250 mL) and washed with $NaHCO_3$ (200 mL). The organic layer was concentrated and diluted with MeOH (20 ml) combined with a saturated solution of $NaHSO_3$ (200 mL) and stirred for 24 hours. The mixture was diluted with DCM and washed with brine (200 mL) and H_2O (200 mL), the organic layer was concentrated *in vacuo*. The residue was dissolved in MeOH (25 mL), DBTO (6.8 g, 27 mmol) was added and the mixture was refluxed at 80 °C for 4 hours, cooled to room temperature and the solvent was removed *in vacuo.* To the solution of the residue in DMF (30 mL) CsF (2.5 g, 18 mmol) and (2-bromomethyl) naphthalene (4.8 g, 22 mmol) were added under Ar protection and the mixture was stirred overnight. The next day the solution was diluted with EtOAc (200 mL) and washed with KF (200 mL)and H_2O (200 mL). The organic layer was filtered through Celite to remove tin residue and concentrated, then TFA (90% aq.) (5 mL) was added dropwise and the mixture was stirred at room temperature for 20 minutes until TLC showed the full consumption of the starting material. The mixture was neutralized with NEt_3, diluted with EtOAc and washed with $NaHCO_3$ and H_2O. The organic layer was dried over Na_2SO_4 and concentrated *in vacuo*. The residue was purified by chromatography (silica gel, eluent: hexane/EtOAc = 9:1→1:1 EtOAc) to give **2** (2.9 g, 50%) as a colorless oil. R_f = 0.4 (toluene/EtOAc 2:1). $[\alpha]_D$ +14 (c = 1, EtOAc). ^1H NMR (600 MHz, $CDCl_3$): δ 7.97 – 7.05 (m, 10H, Ar), 5.18 (d, J = 11.8 Hz, 1H, CH_2 (Nap)), 4.98 (d, J = 11.8 Hz, 1H, CH_2’ (Nap)), 4.59 (d, $J_{1\text{-}2}$ = 9.7 Hz, 1H, *H*-1), 3.91 (dd, $J_{6\text{-}6'}$ = 12.0, $J_{5\text{-}6}$ =3.4 Hz, 1H, *H*-6), 3.81 (dd, $J_{6\text{-}6'}$ = 12.0, $J_{5\text{-}6}$ =4.9 Hz, 1H, *H*-6’), 3.66 (t, $J_{3\text{-}4}$=,J=$_{4\text{-}5}$ = 9.3 Hz, 1H, *H*-4), 3.61 (t, $J_{1\text{-}2}$ =

$J_{2\text{-}3}$ = 9.2 Hz, 1H, *H*-2), 3.51 (t, $J_{2\text{-}3}$= $J_{3\text{-}4}$ = 8.8 Hz, 1H, *H*-3), 3.43 – 3.38 (m, 1H, *H*-5), 2.92 (br s, 1H, O*H*-4), 2.71 (br s, 1H, O*H*-2), 2.44 (s, 3H, S-Ph-C*H*$_3$), 1.34 (s, 9H,S-Ph-C(C*H*$_3$)$_3$).13C NMR (151 MHz, CDCl3) δ 149.77-125.32, (16*C* Ar) 88.88 (*C*-1), 85.12 (*C*-3), 79.33 (*C*-5), 74.92 (*C*H$_2$ (Nap)), 73.02 (*C*-2), 70.01 (*C*-4), 62.70 (*C*-6), 34.48 (S-Ph-*C*(CH$_3$)$_3$), 31.32 (S-Ph-C(*C*H$_3$)$_3$), 20.44 (S-Ph-*C*H$_3$). HRMS (ESI): Calcd m/z for $C_{28}H_{34}O_5S$ $[M+Na]^+$ 505.2019, found 505.2021.

*3.5.3. Methyl [(2-methyl-5-tert-butylphenyl) 3- O-(2-methylnaphthyl)-1-thio-β-D-glucopyranosyl] uronate (**3**)*

3 (2.4 g, 5 mmol) was dissolved in DCM (25 mL), H_2O (5 mL) and TEMPO (155 mg, 1 mmol) were added, followed by slow addition of BAIB (4 g, 12.5 mmol) under ice bath cooling. The reaction mixture was stirred vigorously at 3 °C for 2.5 h until TLC showed full consumption of the starting material to form a single main product. Cold 1 M solution of $Na_2S_2O_3$ (100 mL) was poured into the reaction mixture and stirred for 50 minutes, then diluted with EtOAc and washed with brine and H_2O; the organic layer was dried over Na_2SO_4 and concentrated *in vacuo*. The crude residue was dissolved in DMF (8 mL) and K_2CO_3 (500 mg, 3.6 mmol) and MeI (460 μL, 7.5 mmol) were added. The mixture was stirred for 1 h until TLC (Tol/EtOAc 2:1) showed full consumption of the starting material, then diluted with EtOAc (250 mL) and washed with H_2O (2×250 mL). The organic layer was dried over Na_2SO_4 and concentrated *in vacuo*. The residue was purified by chromatography (silica gel, eluent: hexane/EtOAc = 10:1→4:1) to give **3** (2.3 g, 92%) as colorless oil. R_f= 0.8 (Tol/EtOAc 2:1). $[\alpha]_D$ −50 (c = 1, EtOAc). δ^{1}H NMR (600 MHz, $CDCl_3$) δ 7.98 – 7.09 (m, 10H, Ar), 5.11 (s, 2H, C*H*$_2$ (Nap)), 4.58 (d, $J_{1,2}$ = 9.6 Hz, 1H, *H*-1), 3.97 (t, $J_{3,4}$ = $J_{4,5}$ = 9.3 Hz, 1H, *H*-4), 3.90 (d, $J_{4,5}$ = 9.8 Hz, 1H, *H*-5), 3.84 (s, 3H, O-C*H*$_3$), 3.62 (t, $J_{1,2}$ = $J_{2,3}$ = 9.2 Hz, 1H, *H*-2), 3.56 (t, $J_{2,3}$ = $J_{3,4}$ = 8.7 Hz, 1H, *H*-3), 3.20 (br s, 1H, O*H*-4), 2.68 (br s, 1H, O*H*-2), 2.46 (s, 3H, S-Ph-C*H*$_3$), 1.35 (s, 9H, S-Ph-C(C*H*$_3$)$_3$). ^{13}C NMR (151 MHz, $CDCl_3$) δ 169.44 (*C*=O), 49.68-125.44 (16*C*, Ar), 89.17 (*C*-1), 84.22 (*C*-3), 77.73(*C*-5), 75.12

(CH_2 (Nap)), 71.93 (*C*-2), 71.43 (*C*-4), 52.69 (O-CH_3), 34.47 (S-Ph-*C*(CH_3)$_3$), 31.24 (S-Ph-C(CH_3)$_3$), 20.47 (S-Ph-CH_3). HRMS (ESI): Calcd m/z for $C_{29}H_{34}O_6S$ $[M+Na]^+$533.1968, found 533.1970.

*3.5.4. Methyl [(2-methyl-5-tert-butylphenyl) 3- O-(2-methylnaphthyl)-1-thio-α-D-idopyranosyl] uronate (**4**)*

3 (2.3 g, 4.5 mmol) was dissolved in anhydrous MeOH (20 mL) under Ar protection, activated 3 Å molecular sieves (2 g) were added, followed by freshly prepared 1 M MeONa (8.5 mL). The mixture was stirred for 48 h, filtered through Celite, diluted with EtOAc (200 mL) and washed with 0.5 M HCl (200 mL) and H_2O (200 mL). The organic layer was dried over Na_2SO_4 and concentrated *in vacuo*. To recover any demethylated uronic acid the residue was dissolved in DMF (8 mL) and K_2CO_3 (500 mg, 3.6 mmol) and MeI (230 µL, 2.75 mmol) were added and the mixture was stirred for 1 h until TLC showed no free acid remaining. The mixture was then diluted with EtOAc and washed with H_2O, the organic layer was dried over Na_2SO_4 and concentrated *in vacuo*. The residue was purified by column chromatography (silica gel, eluent: hexane/EtOAc = 6:1→1:1) to give **4** (1.6 g, 50%, R_f = 0.4 (toluene/EtOAc 2:1)) as a colorless oil, as well as recovered **3** (1 g, 31%). $[\alpha]_D$ −141° (c = 1, EtOAc). ^{1}H NMR (600 MHz, $CDCl_3$) δ 7.93 – 7.12 (m, 10H, Ar), 5.55 (s, 1H, *H*-1), 5.36 (d, $J_{4,5}$ = 1.2 Hz, 1H, *H*-5), 4.97 (d, J = 11.9 Hz, 1H, C*H*$_2$ (Nap)), 4.82 (d, J = 11.9 Hz, 1H, C*H*$_2$ (Nap)), 4.29 – 4.22 (m, 2H, *H*-4,*H*-2), 4.04 (s, 1H, O*H*-2), 3.92 (t, $J_{2,3}$=$J_{3,4}$ = 3.0 Hz, 1H, *H*-3), 3.82 (s, 1H, O*H*-4), 3.80 (s, 3H, O-C*H*$_3$), 2.45 (s, 3H, S-Ph-C*H*$_3$), 1.35 (s, 9H, S-Ph-C(C*H*$_3$)$_3$). ^{13}C NMR (151 MHz, $CDCl_3$) δ 170.78 (*C*=O), 149.84 - 124.67 (16*C* Ar), 89.25 (*C*-1), 74.51 (*C*H$_2$ (Nap)), 72.52 (*C*-3), 69.14 - 68.49 (*C*-2, *C*-4, *C*-5), 52.47 (O-*C*H$_3$), 34.50 (S-Ph-*C*(CH_3)$_3$), 31.31 (S-Ph-C(*C*H$_3$)$_3$), 20.34 (S-Ph-*C*H$_3$). HRMS (ESI): Calcd m/z for $C_{29}H_{34}O_6S$ $[M+Na]^+$ 533.1968, found 533.1971.

*3.5.5. Methyl [(2-methyl-5-tert-butylphenyl) 2-O-benzoyl-3- O-(2-methylnaphthyl)-4-O-tert-butyldimethylsilyl-1-thio-α-D-idopyranosyl] uronate (**5**)*

To a solution of **4** (600 mg, 1,2 mmol) in toluene (15 mL) DBTO (440 mg, 1.8 mmol) was added and the mixture was refluxed for 40 minutes with continuous removal of water (Dean-Stark apparatus), then concentrated to half-volume and cooled to room temperature. BzCl (150 µL, 1.3 mmol) was added and the mixture was stirred for 10 minutes until TLC showed the complete transformation of the starting material. MeOH was added to the mixture, which was then stirred at 40 °C for 30 minutes and concentrated *in vacuo*. The residue was purified by column chromatography (silica gel, eluent: hexane/EtOAc = 10:1→4:1) to give the 2-*O*-benzoylated product (595 mg, 0.97 mmol). This was then dissolved in DCM under Ar protection, NEt_3 (1.4 mL, 10 mmol) and *tert*-butyldimethylsilyl trifluoromethanesulfonate (445 µL, 1.94 mmol) were added under ice bath cooling. The mixture was stirred at room temperature overnight, then diluted with EtOAc (100 mL) and washed with $NaHCO_3$ (150 mL) and H_2O (150 mL). The organic layer was dried over Na_2SO_4, concentrated *in vacuo* and purified by column chromatography (silica gel, eluent: hexane/EtOAc = 25:1→10:1) to yield **5** (465 mg, 54%) as a colorless oil. R_f = 0.9 (hexane/EtOAc 2:1) $[\alpha]_D$ −20 (c = 1, EtOAc) ^{1}H NMR (600 MHz, $CDCl_3$) δ 8.18 – 7.05 (m, 15H, Ar), 5.74 (d, $J_{1,2}$ = 2.8 Hz, 1H, *H*-1), 5.48 (t, $J_{1,2}$ =$J_{2,3}$= 3.2 Hz, 1H, *H*-2), 5.32 (d, $J_{4,5}$ = 2.8 Hz, 1H, *H*-5), 5.11 (d, J = 12.0 Hz, 1H, CH_2 (Nap)), 4.93 (d, J = 12.0 Hz, 1H, CH_2 (Nap)), 4.20 (t, $J_{3,4}$ = $J_{4,5}$= 3.3 Hz, 1H, *H*-4), 3.98 (t, $J_{2,3}$ = $J_{3,4}$ = 3.7 Hz, 1H, *H*-3), 3.80 (s, 3H, O-CH_3), 2.42 (s, 3H, S-Ph-CH_3), 1.33 (s, 9H, S-Ph-C(CH_3)$_3$), 0.70 (s, 9H, Si-C(CH_3)$_3$), -0.09 (s, 3H, Si-CH_3), -0.31 (s, 3H, Si-CH_3’). ^{13}C NMR (151 MHz, $CDCl_3$) δ 169.95-165.85 (2*C*=O), 149.63 - 125.28 (16*C* Ar), 85.95 (*C*-1), 74.89 (*C*-3), 72.97 (*C*H_2 (Nap)), 70.79 (*C*-5), 69.97 (*C*-2), 69.29 (*C*-4), 51.99 (O-*C*H_3), 31.29 (S-Ph-C(*C*H_3)$_3$), 25.52 (Si-C(*C*H_3)$_3$), 20.40 (S-Ph-*C*H_3), -4.42 (Si-*C*H_3), -5.78 (Si-*C’*H_3). HRMS (ESI): Calcd m/z for $C_{42}H_{52}O_7SSi$ $[M+Na]^+$ 751.3095, found 751.3094.

*3.5.6. Methyl [(3-azidopropyl) 2-O-benzoyl-3- O-(2-methylnaphthyl)-4-O-tert-butyldimethylsilyl-α-D-idopyranosyl] uronate (**6**)*

To a solution of **5** (465 mg, 0.63 mmol)) in DCM 3-chloroperbenzoic acid (235 mg, 1.35 mmol) was added at −50 °C under Ar protection, the mixture was stirred for 50 minutes at −50 °C to −10 °C, until TLC showed the full consumption of the starting material, then diluted with EtOAc (100 mL) and washed with 1 M $Na_2S_2O_3$ (150 mL) and H_2O (150 mL). The organic layer was dried over Na_2SO_4, concentrated *in vacuo* and purified by column chromatography (silica gel, eluent: hexane/EtOAc = 10:1→3:1) to give a mixture of diastereomeric sulfoxide glycosyl donors (410 mg, 0.55 mmol, R_f = 0.7 (hexane/EtOAc 2:1)), which were immediately used for glycosylation. The sulfoxide donors were dissolved in DCM (12 mL), activated AW300 molecular sieves (420 mg), 2,6-di-*tert*-butyl-4-methylpyridine (412 mg, 1.65 mmol) and 3-azido-1-propanol (404 µL, 4.4 mmol) were added and the mixture was stirred for 30 minutes, after which Tf_2O (242 µL, 1.43 mmol) was added at room temperature. After a few minutes TLC showed complete consumption of the starting material, the mixture was neutralized with NEt_3, diluted with EtOAc (100 mL), filtered through Celite and washed with $NaHCO_3$ (100 mL) and H_2O (100 mL). The organic layer was dried over Na_2SO_4 and concentrated *in vacuo*.. Purification by column chromatography (silica gel, eluent: hexane/EtOAc = 15:1→6:1) gave **6** (290 mg, 70%) as a colorless oil. R_f = 0.9 (hexane/EtOAc 2:1) $[\alpha]_D$ −23 (c = 1, EtOAc). ^{1}H NMR (600 MHz, $CDCl_3$) δ 8.10 – 7.26 (m, 12H, Ar), 5.29 (d, $J_{1,2}$ = 3.0 Hz, 1H, *H*-1), 5.19 (t, $J_{1,2}$ = $J_{2,3}$ = 3.1 Hz, 1H, *H*-2), 5.04 (d, J = 12.0 Hz, 1H, C*H*$_2$ (Nap)), 4.91 (d, J = 12.0 Hz, 1H, C*H*$_2$ (Nap)), 4.80 (d, $J_{4,5}$ = 3.1 Hz, 1H, *H*-5), 4.16 (t, $J_{3,4}$ = $J_{4,5}$ = 3.5 Hz, 1H, *H*-4), 3.96 – 3.93 (m, 1H, O-C*H*$_2$-CH_2-CH_2-N_3), 3.88 (t, $J_{2,3}$ = $J_{3,4}$.= 6 Hz, 1H, *H*-3), 3.82 (s, 3H, O-C*H*$_3$), 3.66 – 3.62 (m, 1H, O-C*H’*$_2$-CH_2-CH_2-N_3), 3.37 (t, J = 6.7 Hz, 2H, O-CH_2-CH_2-C*H*$_2$-N_3), 1.92 – 1.86 (m, 2H, O-CH_2-C*H*$_2$-CH_2-N_3), 0.74 (s, 9H, Si-C(C*H*$_3$)$_3$), -0.08 (s, 3H, Si-C*H*$_3$), -0.29 (s, 3H, Si-C*H*$_3$’). ^{13}C NMR (151 MHz, $CDCl_3$) δ 170.23-165.78 (2*C*=O), 135.15-125.88 (16*C*, Ar), 98.72 (*C*-1), 75.59 (*C*-3), 72.58 (*C*H_2 (Nap)), 70.34 (*C*-5), 69.39 (*C*-2), 69.03 (*C*-4), 65.25 (O-*C*H_2-CH_2-CH_2-N_3), 52.06

(O-CH_3), 48.18 (O-CH_2-CH_2-CH_2-N_3), 28.89 (O-CH_2-CH_2-CH_2-N_3), 25.53 (Si-C(CH_3)$_3$), -4.43 (Si-CH_3), -5.79 (Si-$C'H_3$). HRMS (ESI): Calcd m/z for $C_{34}H_{43}N_3O_8Si$ $[M+Na]^+$ 672.2712, found 672.2704.

3.5.7. (3-Aminopropyl) -α-D-idopyranosyluronic sodium salt (7)

Compound **6** (40 mg, 0.06 mmol) was deprotected following standard procedures A1 (purification by silica gel chromatography, eluent: hexane:EtOAc 5:1→1:1), then E1, E2 and F, and purified by gel chromatography on a Toyopearl HW-40S column, followed by lyophilization to give compound **7** (6.7 mg, 40%) as an amorphous white solid. $[\alpha]_D$ −45 (c = 0.268, H_2O). ^1H NMR (600 MHz, D_2O) δ 4.76 (d, $J_{1,2}$ = 5.0 Hz, 1H, *H*-1), 4.39 (d, $J_{4,5}$ = 4.1 Hz, 1H, *H*-5), 3.99 – 3.92 (m, 1H, O-CH_2-CH_2-CH_2-NH_2), 3.84 (dd, $J_{3,4}$ = 6.2, $J_{4,5}$ =.4 Hz, 1H, *H*-4), 3.79 – 3.73 (m, 1H, O-CH_2'-CH_2-CH_2-NH_2), 3.71 (t, J = 6.7 Hz, 1H, *H*-3), 3.49 – 3.45 (m, 1H, *H*-2), 3.15 – 3.12 (m, 2H, O-CH_2-CH_2-CH_2-NH_2), 2.07 – 1.91 (m, 2H, O-CH_2-CH_2-CH_2-NH_2). ^{13}C NMR (151 MHz, D_2O) δ 100.84 (*C*-1), 72.23 (*C*-3), 71.10-70.96 (*C*-2, *C*-4, *C*-5), 67.43 (O-CH_2-CH_2-CH_2-NH_2), 38.32 (O-CH_2-CH_2-CH_2-NH_2), 26.90 (O-CH_2-CH_2-CH_2-NH_2). HRMS (ESI): Calcd m/z for $C_9H_{17}NO_7$ $[M+H]^+$ 252.1078, found 252.1088.

*3.5.8. (3-Aminopropyl) 2-O-sulfo-α-D-idopyranosyluronic sodium salt (**8**)*

Compound **6** (40 mg, 0.06 mmol) was selectively deprotected following standard procedure C and purified by column chromatography (silica gel, eluent: hexane/EtOAc 8:1→4:1). The product was sulfated according to standard procedure D and purified by silica gel chromatography (eluent DCM/MeOH 40:1→5:1), and then subjected to final deprotection according to procedures A2 (purification by silica gel chromatography, eluent DCM/MeOH 50:1→10:1), E1 and F. Purification by gel filtration using a Toyopearl HW-40S column followed by lyophilization gave compound **8** (15.8 mg, 68%) as an amorphous white solid. $[\alpha]_D$ −23 (c = 0.658, H_2O). ^1H NMR (600 MHz, D_2O) δ 5.04 (d, $J_{1,2}$ = 2.2 Hz, 1H, *H*-1), 4.45 (d, $J_{4,5}$ = 2.6 Hz, 1H), 4.20 (dd, $J_{2,3}$ = 4.0, $J_{1,2}$= 2.5 Hz, 1H, *H*-2), 4.03 (t, $J_{2,3}$ = $J_{3,4}$ =4.2 Hz, 1H, *H*-3), 3.99

– 3.96 (m, 1H, *H*-4), 3.95 – 3.89 (m, 1H, O-CH_2-CH_2-CH_2- NH_2), 3.74 – 3.66 (m, 1H, O-CH_2'-CH_2-CH_2- NH_2), 3.15 (t, J = 6.5 Hz, 2H, O-CH_2-CH_2-CH_2- NH_2), 2.05 – 1.91 (m, 2H, O-CH_2-CH_2-CH_2- NH_2). ^{13}C NMR (151 MHz, D_2O) δ 99.08 (*C*-1), 75.42 (*C*-2), 69.83 (*C*-3, *C*-4), 69.20 (*C*-5), 67.23 (O-*C*H_2-CH_2-CH_2- NH_2), 38.83 (O-CH_2-CH_2-*C*H_2- NH_2), 26.70(O-CH_2-*C*H_2-CH_2-NH_2). HRMS (ESI): Calcd m/z for $C_9H_{17}NO_{10}S$ $[M-H]^-$ 330.0500, found 330.0499.

*3.5.9. (3-Aminopropyl) 3-O-sulfo-α-D-idopyranosyluronic sodium salt (**9**)*

Compound **6** (16 mg, 0.03 mmol) was treated according to standard procedure B and purified by silica gel chromatography (eluent hexane/EtOAc, 10:1 → 4:1). The selectively deprotected product was sulfated according to standard procedure D and purified by silica gel chromatography (eluent DCM/MeOH 40:1→5:1), then subjected to final deprotection according to procedures A2 (purification by silica gel chromatography, eluent DCM/MeOH 50:1→10:1), E1, E2 and F. Purification by gel filtration using a Toyopearl HW-40S column followed by lyophilization gave compound **9** (3 mg, 32%). $[\alpha]_D$ −40 (c = 0.125, H_2O). ^{1}H NMR (600 MHz, D_2O) δ 4.92 (s, 1H, *H*-1), 4.50 – 4.46 (m, 1H, *H*-3), 4.39 (d, $J_{4,5}$ = 2.0 Hz, 1H, *H*-5), 4.22 – 4.18 (m, 1H, *H*-4), 3.97 – 3.93 (m, 1H, O-CH_2-CH_2-CH_2-NH_2), 3.88 – 3.87 (m, 1H, *H*-2), 3.73 – 3.70 (m, 1H, O-CH_2'-CH_2-CH_2-NH_2), 3.18 – 3.14 (m, 2H, O-CH_2-CH_2-CH_2-NH_2), 2.03 – 1.94 (m, 2H, O-CH_2-CH_2-CH_2-NH_2). ^{13}C NMR (151 MHz, D_2O) δ 100.50 (*C*-1), 75.63 (*C*-3), 68.85 (*C*-5), 67.88 (*C*-4, O-*C*H_2-CH_2-CH_2-NH_2), 66.94 (*C*-2), 39.56 (O-CH_2-CH_2-*C*H_2-NH_2), 26.94 (O-CH_2-*C*H_2-CH_2-NH_2). HRMS (ESI): Calcd m/z for $C_9H_{17}NO_{10}S$ $[M-H]^-$ 330.0500, found 330.0486.

*3.5.10. (3-Aminopropyl) 3,4-di-O-sulfo-α-D-idopyranosyluronic sodium salt (**10**)*

Compound **6** (45 mg, 0.07 mmol) was selectively deprotected following procedures A1 (purification by silica gel chromatography, eluent hexane/EtOAc 5:1 → 1:1) and B (purification by silica gel chromatography, eluent hexane/EtOAc 4:1 → 1:2). The product was sulfated according to standard procedure D and purified by silica gel chromatography (eluent

DCM/MeOH 40:1→5:1), then subjected to final deprotection according to procedures E1, E2 and F. Purification by gel filtration using a Toyopearl HW-40S column followed by lyophilization gave compound **10** (14 mg, 42%) as an amorphous white solid. $[\alpha]_D$ −44 (c = 0.588, H_2O). ^1H NMR (600 MHz, D_2O) δ 5.01 – 4.98 (m, 1H, *H*-3), 4.94 (s, 1H, *H*-1), 4.82 – 4.80 (m, 1H, *H*-4), 4.48 (d, $J_{4,5}$ = 1.9 Hz, 1H, *H*-5), 3.94 – 3.89 (m, 2H, *H*-2, O-CH_2-CH_2-CH_2-N_3), 3.74 – 3.68 (m, 1H, O-CH_2'-CH_2-CH_2-NH_2), 3.19 – 3.17 (m, 2H, O-CH_2-CH_2-CH_2-NH_2), 2.05 – 1.93 (m, 2H, O-CH_2-CH_2-CH_2-NH_2). ^{13}C NMR (151 MHz, D_2O) δ 100.38 (*C*-1), 73.15 (*C*-4), 72.81 (*C*-3), 67.87 (O-*C*H_2-CH_2-CH_2-NH_2), 67.25 (*C*-5), 66.47 (*C*-2), 39.65 (O-CH_2-CH_2-*C*H_2-NH_2), 26.65 (O-CH_2-*C*H_2-CH_2-NH_2). HRMS (ESI): Calcd m/z for $C_9H_{16}NO_{13}S_2Na$ $[M-H]^-$ 431.9888, found 431.9876.

*3.5.11. (3-Aminopropyl) 2,3,4-tri-O-sulfo-α-D-idopyranosyluronic sodium salt (**11**)*

Compound **6** (29 mg, 0.12 mmol) was selectively deprotected following procedures A1 (purification by silica gel chromatography, eluent hexane/EtOAc 5:1 → 1:1) and B (purification by silica gel chromatography, eluent hexane/EtOAc 4:1 → 1:2). The product was then subjected to benzoyl cleavage according to procedure C and without purification sulfated following procedure D, then purified by gel-chromatography on a Sephadex G-15. The purified product was then subjected to final deprotection following procedures E1 and F, purified by gel filtration using a Toyopearl HW-40S column and lyophilized to give **11** (6.5 mg, 25%) as an amorphous white solid. $[\alpha]_D$ −23 (c = 0.21, H_2O). ^1H NMR (600 MHz, D_2O) δ 5.16 – 5.14 (m, 1H, *H*-3), 5.12 (br s, 1H, *H*-1), 4.80 – 4.79 (m, 1H, *H-4*), 4.50 – 4.49 (m, 2H, *H*-2, *H-5*), 3.95 – 3.91 (m, 2H, *H*-2, O-CH_2-CH_2-CH_2-NH_2), 3.75 – 3.71 (m, 1H, O-CH_2'-CH_2-CH_2-NH_2), 3.20 – 3.17 (m, 2H, O-CH_2-CH_2-CH_2-NH_2), 2.06 – 1.93 (m, 2H, O-CH_2-CH_2-CH_2-NH_2). ^{13}C NMR (151 MHz, D_2O) δ 98.51 (*C*-1), 72.31 (*C*-4), 71.46 (*C*-3), 71.00 (*C*-2), 67.88 (O-*C*H_2-CH_2-CH_2-NH_2), 66.87 (*C*-5), 39.53 (O-CH_2-CH_2-*C*H_2-NH_2), 26.68 (O-CH_2-*C*H_2-CH_2-NH_2). HRMS(ESI): Calcd m/z for $C_9H_{15}NO_{16}S_3Na_2$ $[M-H]^-$ 533.9276, found 533.9275.

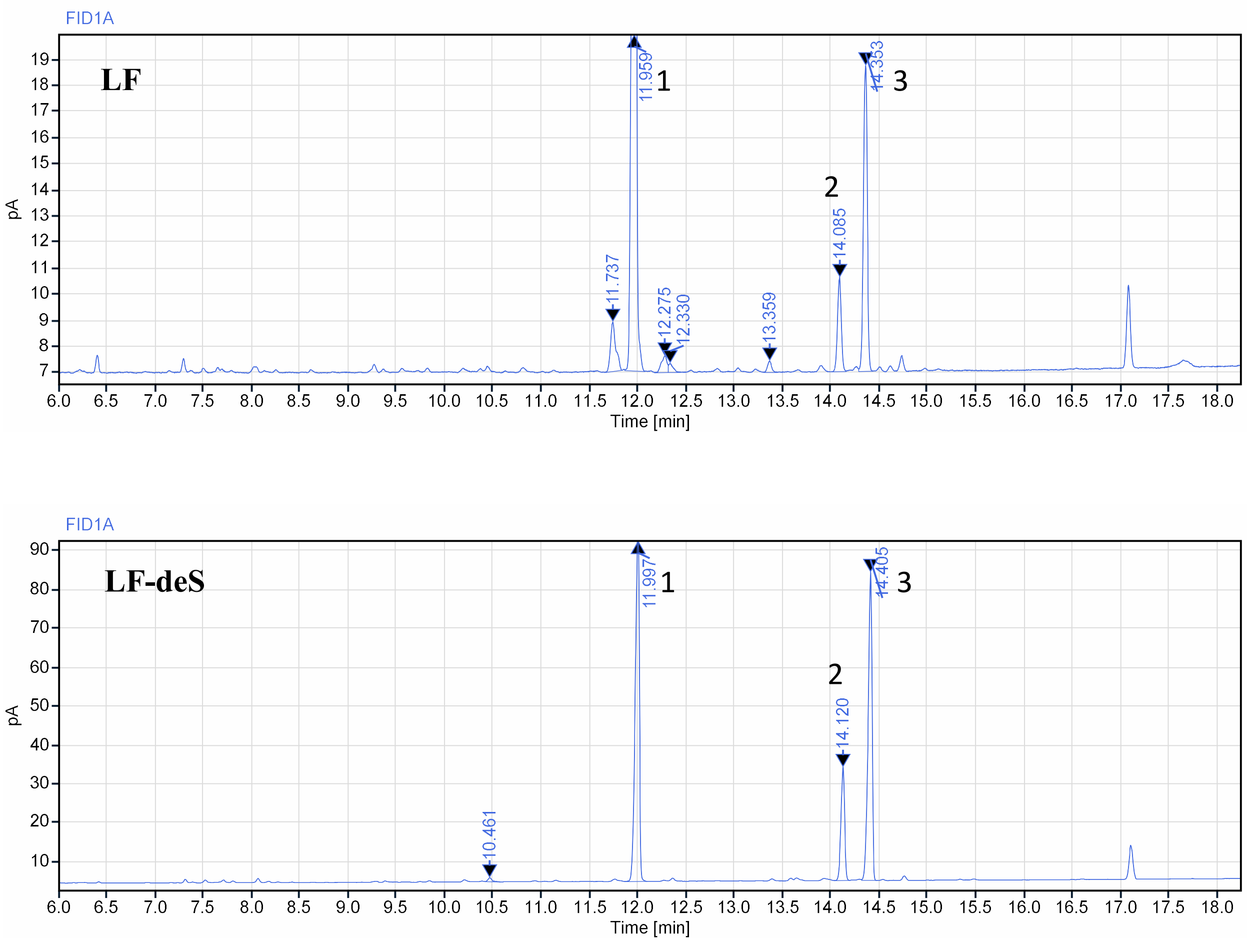

Figure S1. GLC analysis of aminosugar composition of **LF** and **LF-deS**.
1 –myo-inositol acetate (internal standard), 2 – glucosaminitol acetate, 3 – galactosaminitol acetate.

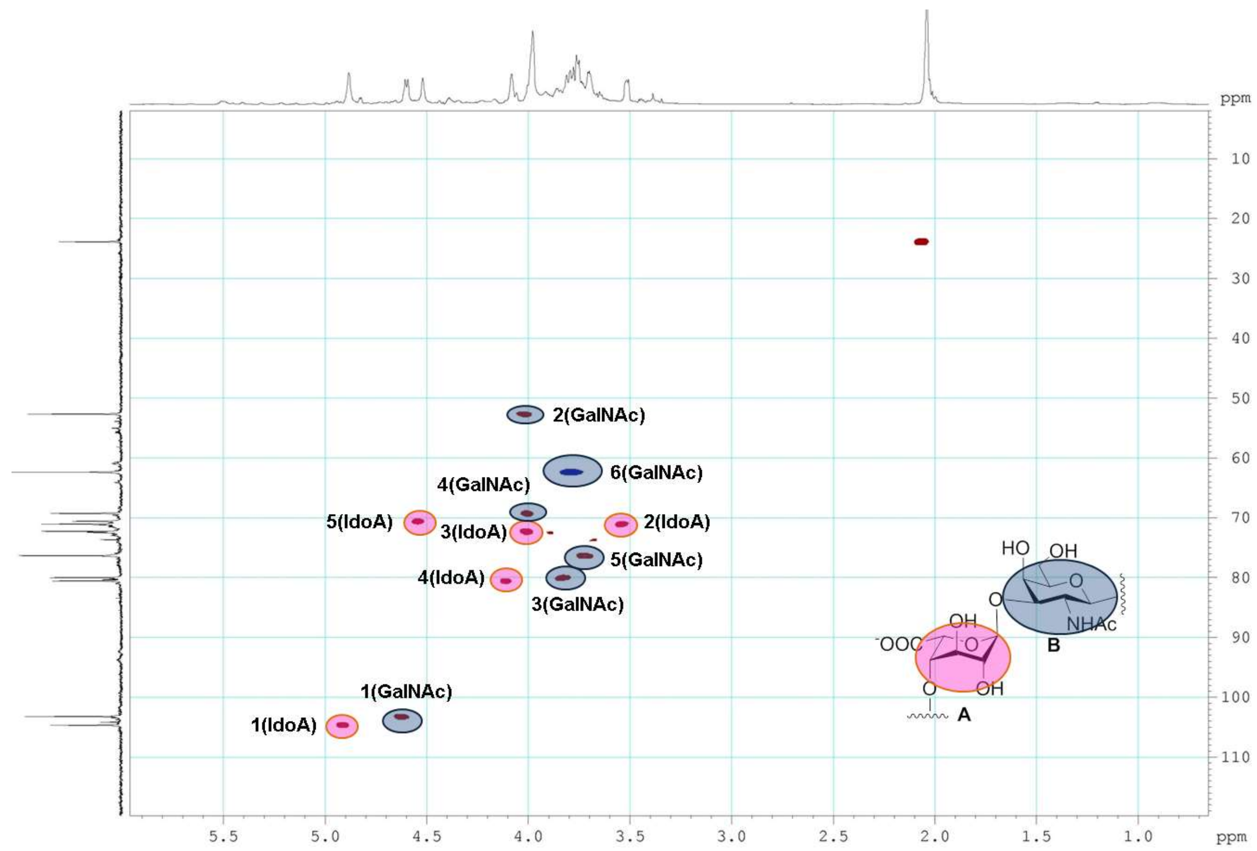

Figure S2. The HSQC NMR spectrum of **LF-deS**.

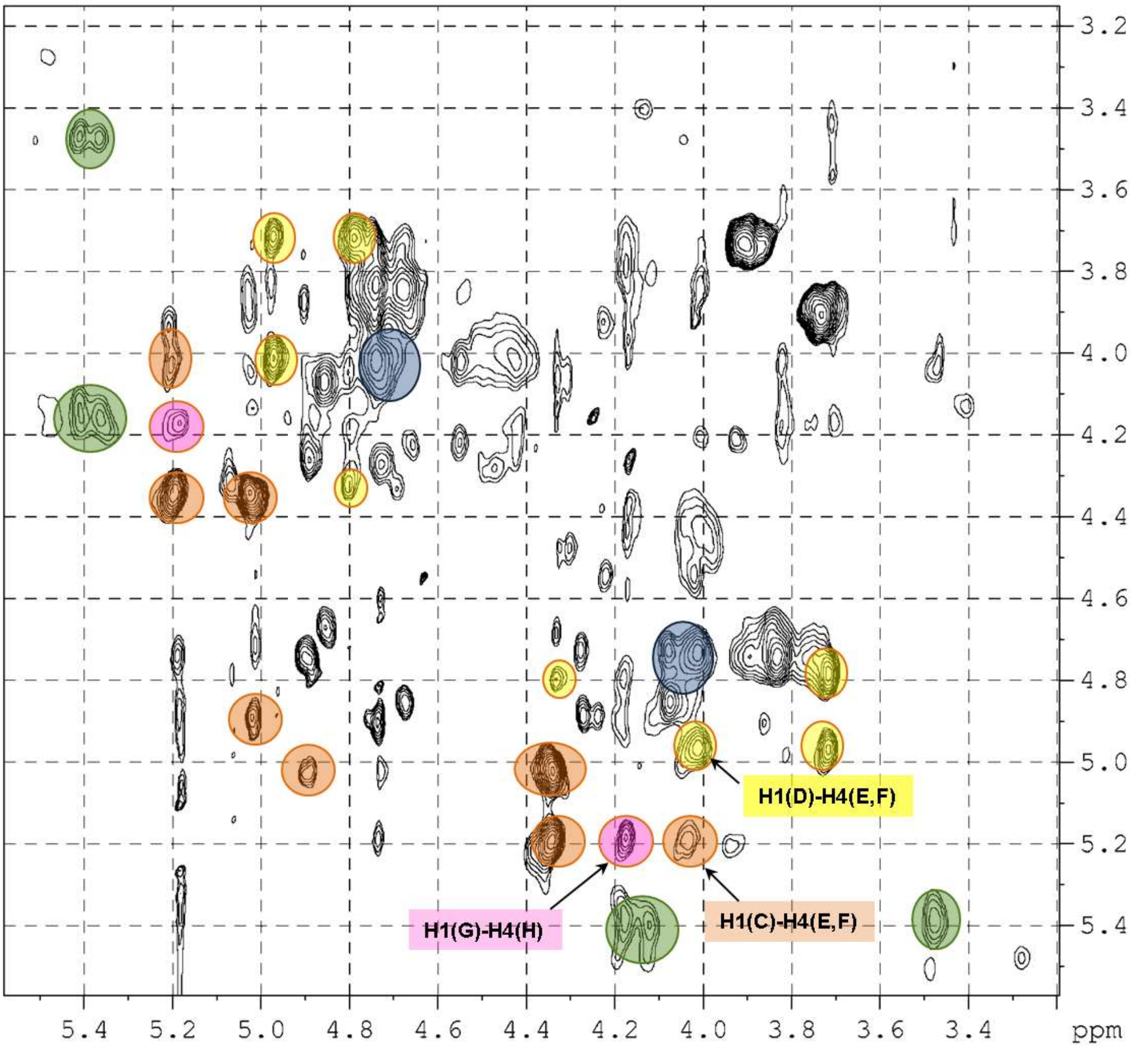

Figure S3. The ROESY NMR spectrum of **LF**.

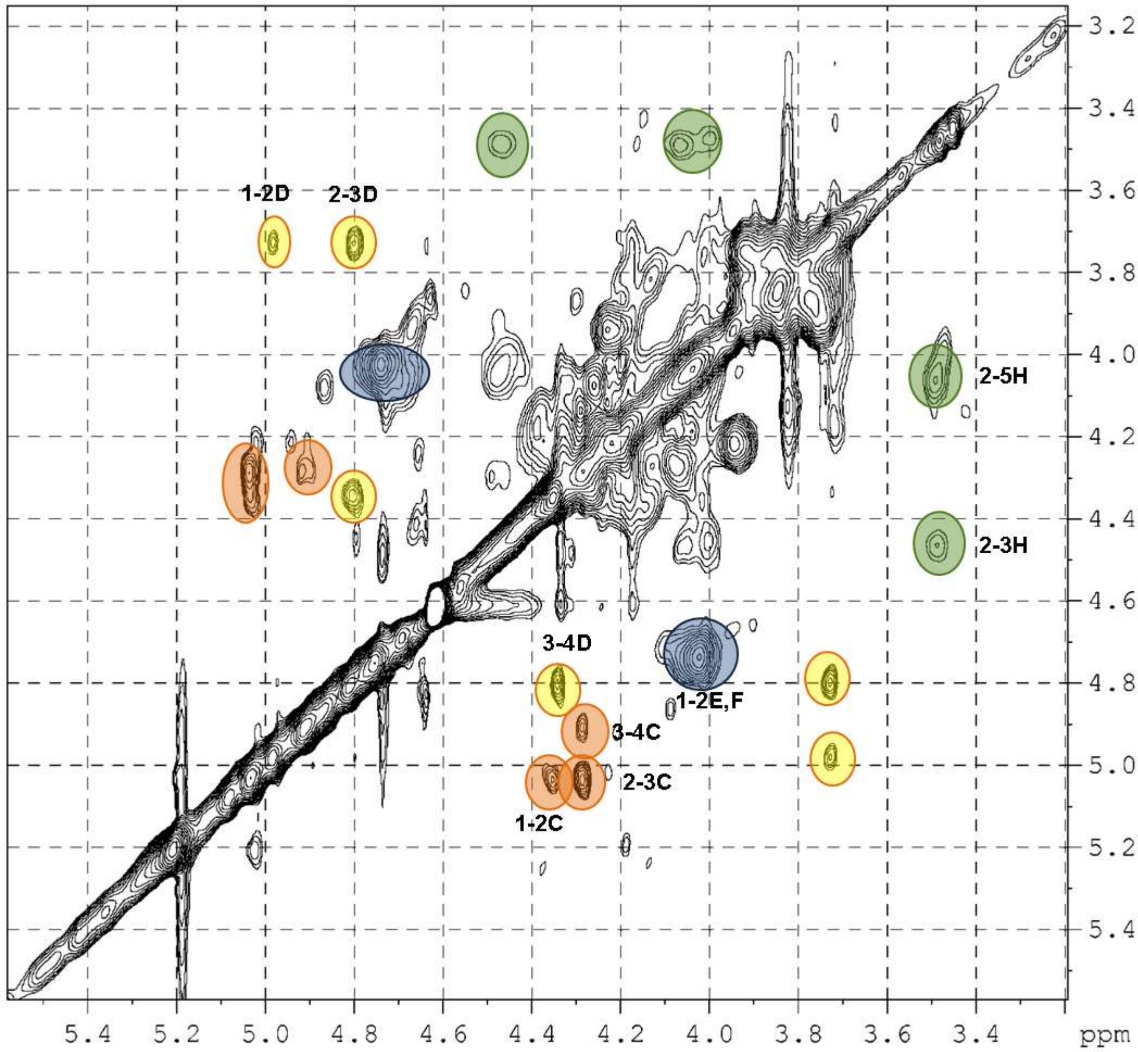

Figure S4. The TOCSY NMR spectrum of **LF**.

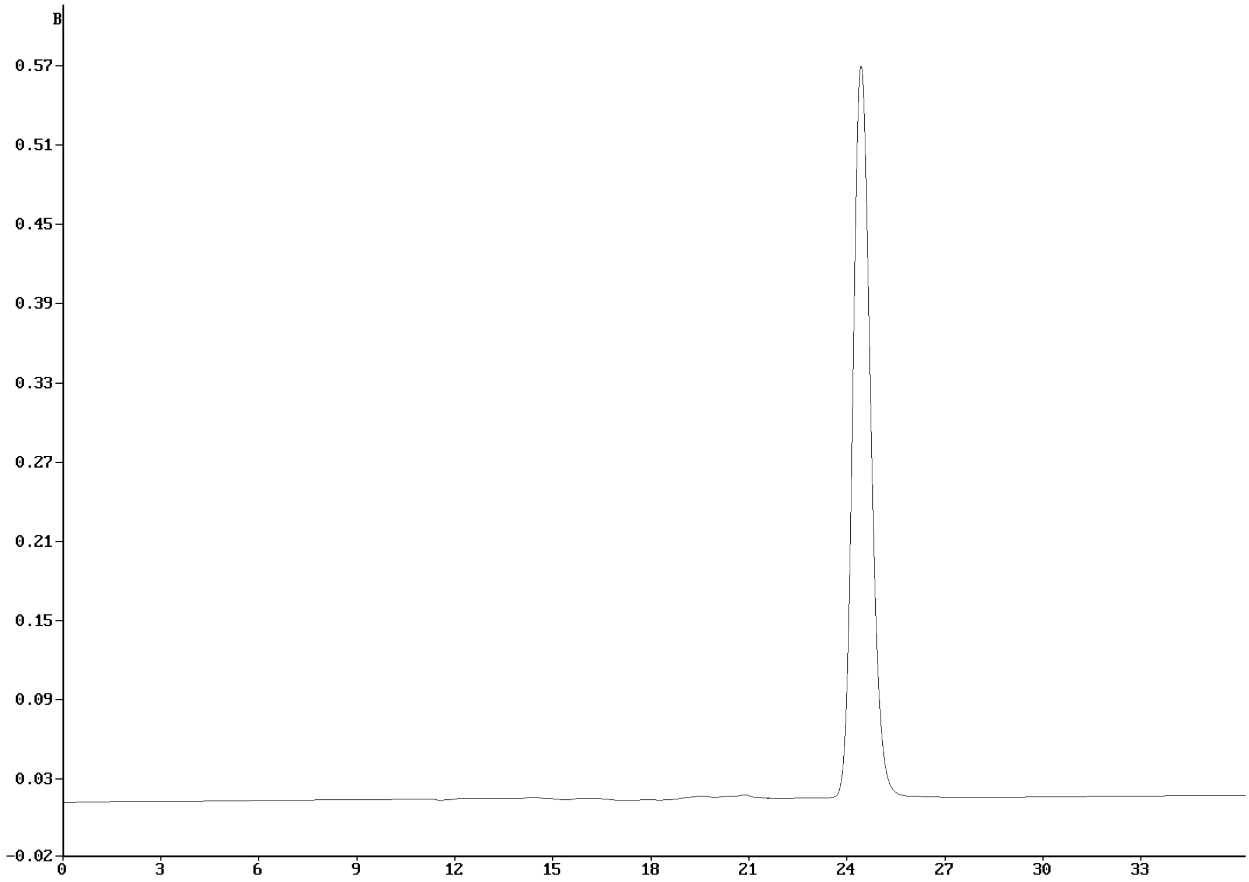

Figure S5. The chromatograms of polysaccharide preparation **LF** on TSK 2000 SW_{XL} column (Toyo Soda, Japan, 7.5×300 mm) calibrated using pullulans (Fluka) at flow rate of 0.75 mL/min by elution with 1 M NaCl in PBS.

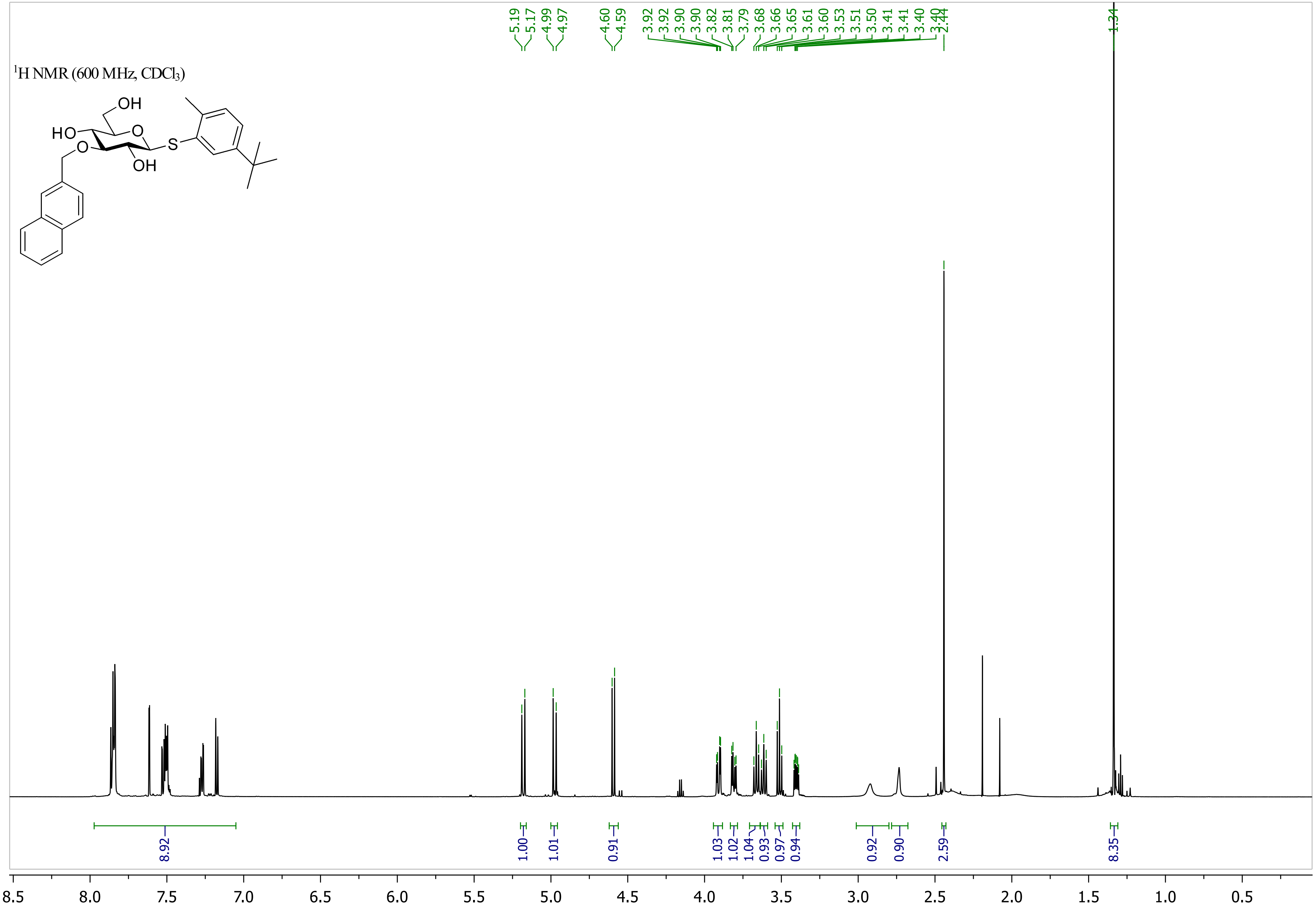

1H NMR (600 MHz, CDCl3)
OH
HO
O
O
S
OH
5.19
5.17
4.99
4.97
4.60
4.59
3.92
3.92
3.90
3.90
3.82
3.81
3.79
3.68
3.66
3.65
3.61
3.60
3.53
3.51
3.50
3.41
3.41
3.40
3.40
2.44
1.34
8.92
1.00
1.01
0.91
1.03
1.02
1.04
0.93
0.97
0.94
0.92
0.90
2.59
8.35
8.5
8.0
7.5
7.0
6.5
6.0
5.5
5.0
4.5
4.0
3.5
3.0
2.5
2.0
1.5
1.0
0.5

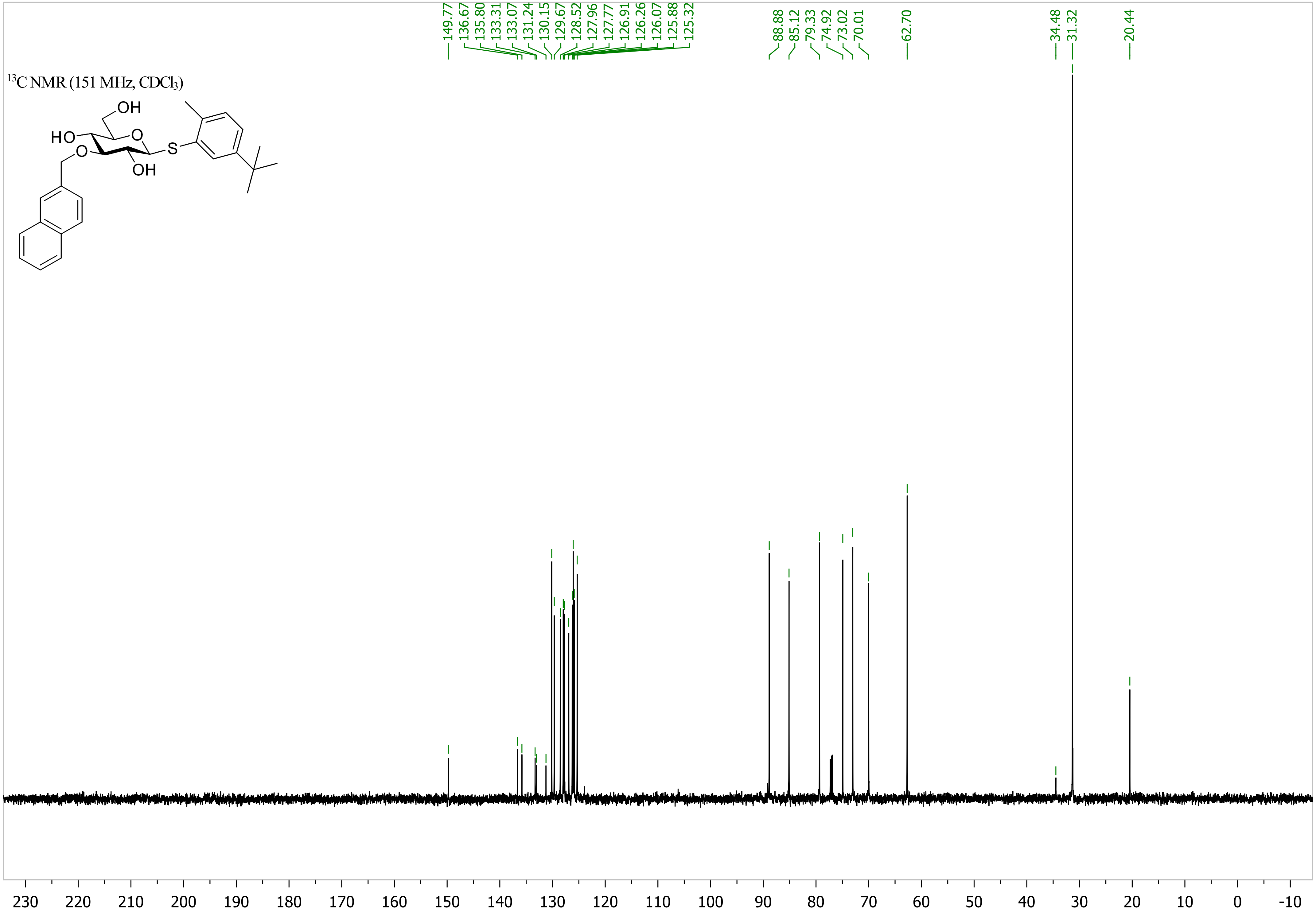

^{13}C NMR (151 MHz, CDCl$_3$)
OH
HO
O
S
O
OH
149.77
136.67
135.80
133.31
133.07
131.24
130.15
129.67
128.52
127.96
127.77
126.91
126.26
126.07
125.88
125.32
88.88
85.12
79.33
74.92
73.02
70.01
62.70
34.48
31.32
20.44
230 220 210 200 190 180 170 160 150 140 130 120 110 100 90 80 70 60 50 40 30 20 10 0 -10

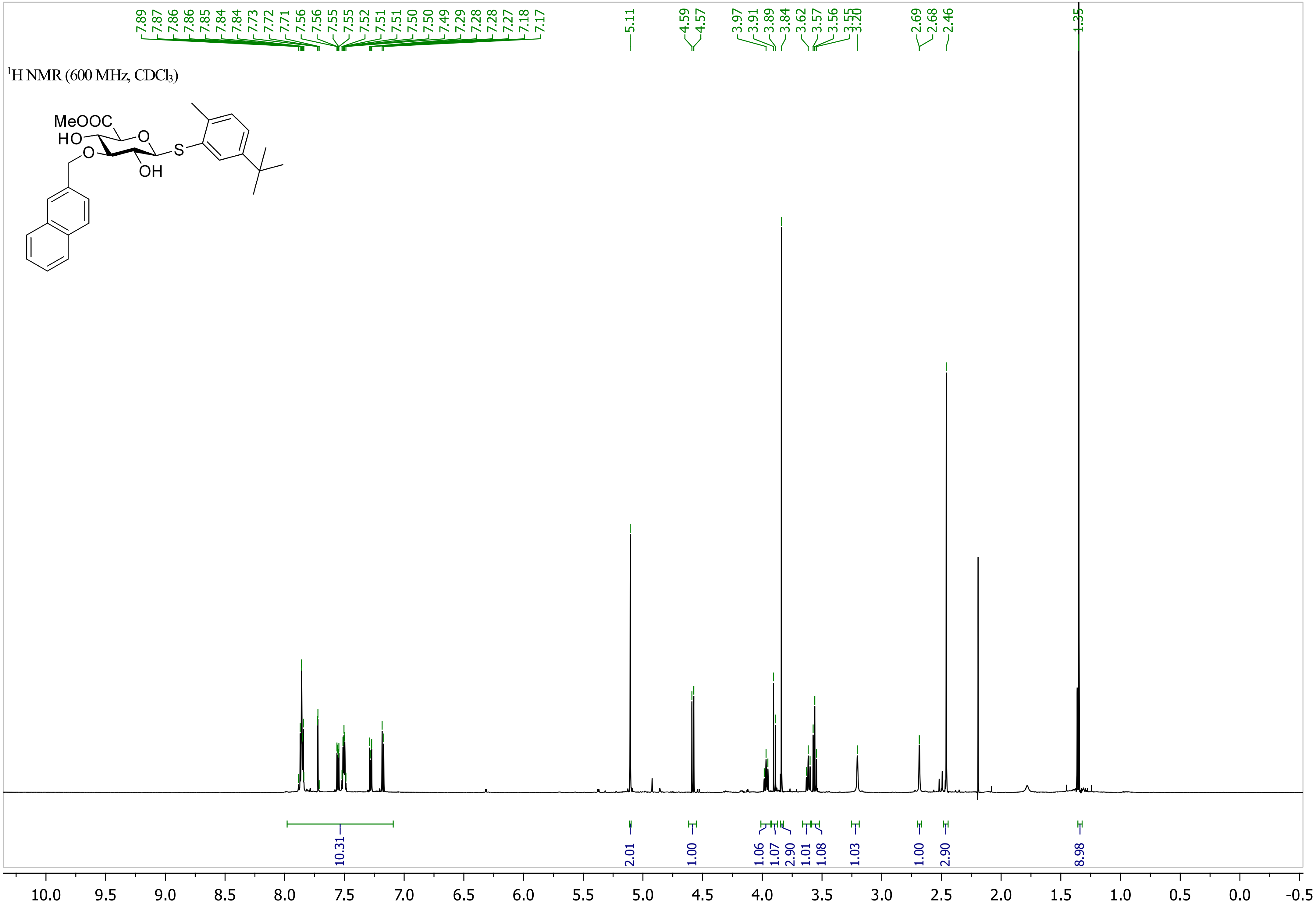

^{1}H NMR (600 MHz, CDCl$_3$)
MeOOC
HO
O
O
S
OH
7.89 7.87 7.86 7.86 7.85 7.84 7.84 7.73 7.72 7.71 7.56 7.56 7.55 7.55 7.52 7.51 7.51 7.50 7.50 7.49 7.29 7.28 7.28 7.27 7.18 7.17
5.11
4.59 4.57
3.97 3.91 3.89 3.84 3.62 3.57 3.56 3.55 3.20
2.69 2.68 2.46
1.35
10.31
2.01
1.00
1.06 1.07 2.90 1.01 1.08
1.03
1.00 2.90
8.98
10.0 9.5 9.0 8.5 8.0 7.5 7.0 6.5 6.0 5.5 5.0 4.5 4.0 3.5 3.0 2.5 2.0 1.5 1.0 0.5 0.0 -0.5

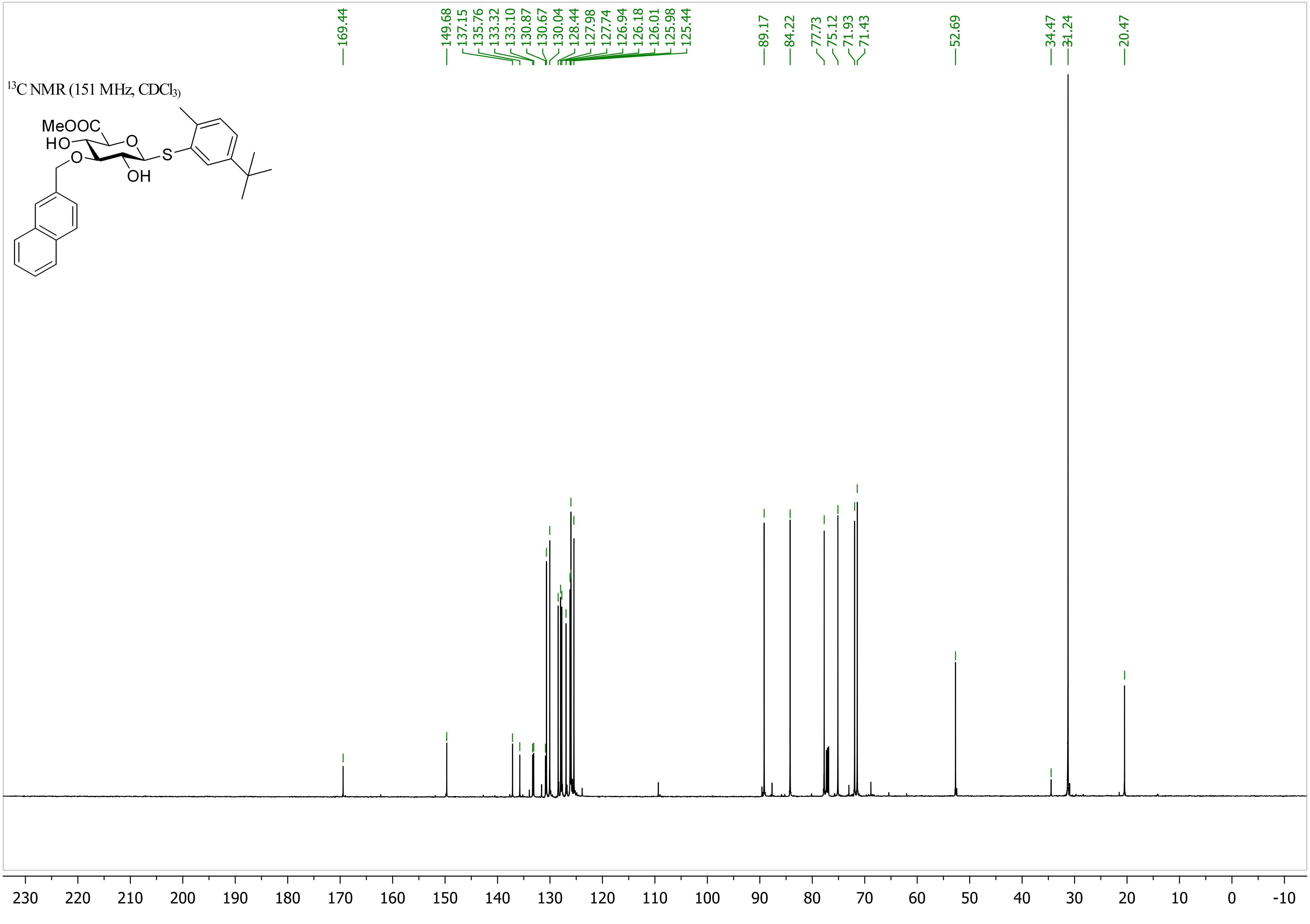

13C NMR (151 MHz, CDCl3)
MeOOC
HO
O
O
S
OH
169.44
149.68
137.15
135.76
133.32
133.10
130.87
130.67
130.04
128.44
127.98
127.74
126.94
126.18
126.01
125.98
125.44
89.17
84.22
77.73
75.12
71.93
71.43
52.69
34.47
31.24
20.47
230 220 210 200 190 180 170 160 150 140 130 120 110 100 90 80 70 60 50 40 30 20 10 0 -10

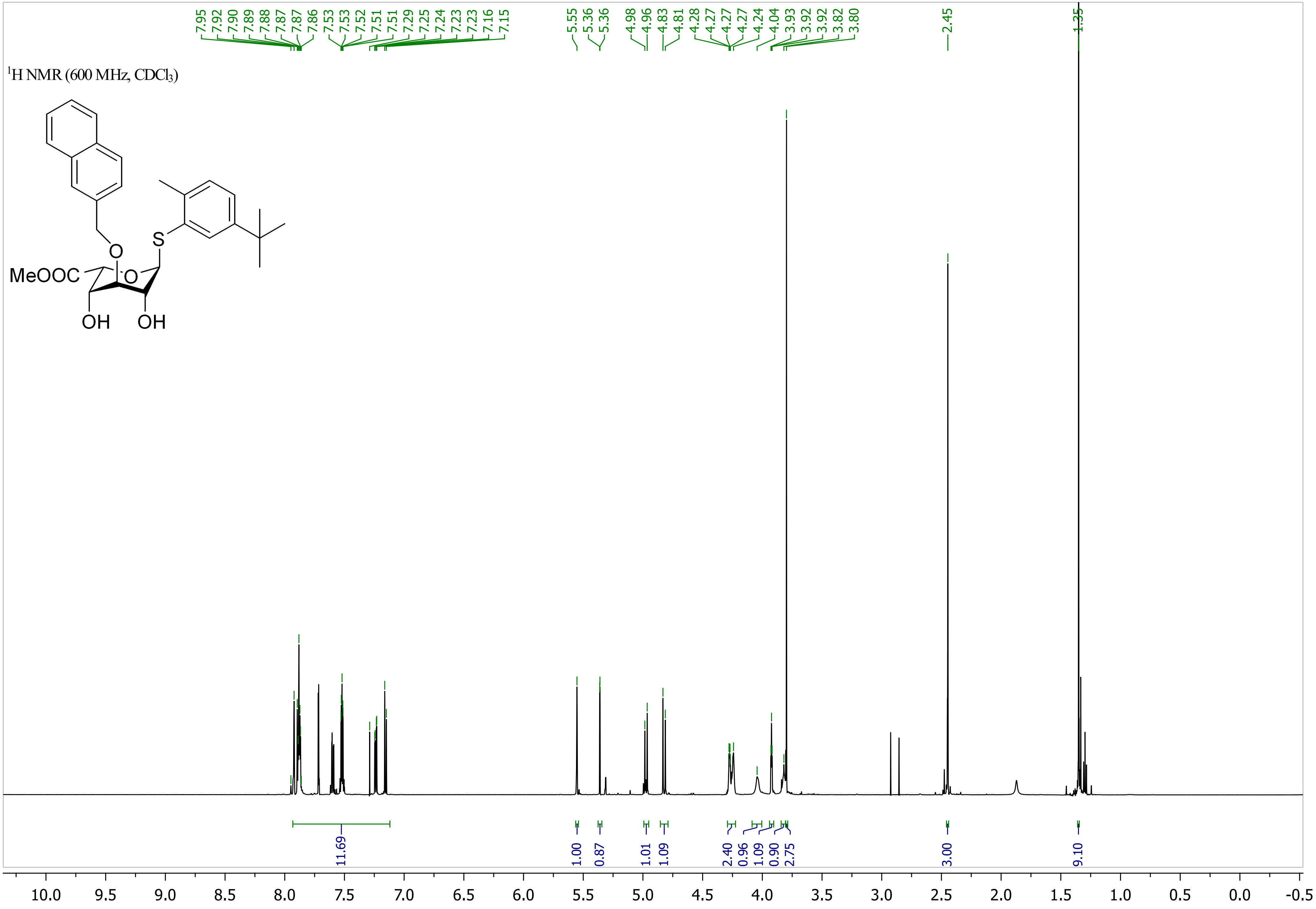

1H NMR (600 MHz, CDCl3)
MeOOC
O
O
S
OH
OH
7.95 7.92 7.90 7.89 7.88 7.87 7.87 7.86 7.53 7.53 7.52 7.51 7.51 7.29 7.25 7.24 7.23 7.23 7.16 7.15
5.55 5.36 5.36 4.98 4.96 4.83 4.81 4.28 4.27 4.27 4.27 4.24 4.04 3.93 3.92 3.92 3.82 3.80
2.45
1.35
11.69
1.00 0.87 1.01 1.09 2.40 0.96 1.09 0.90 2.75
3.00
9.10
10.0 9.5 9.0 8.5 8.0 7.5 7.0 6.5 6.0 5.5 5.0 4.5 4.0 3.5 3.0 2.5 2.0 1.5 1.0 0.5 0.0 -0.5

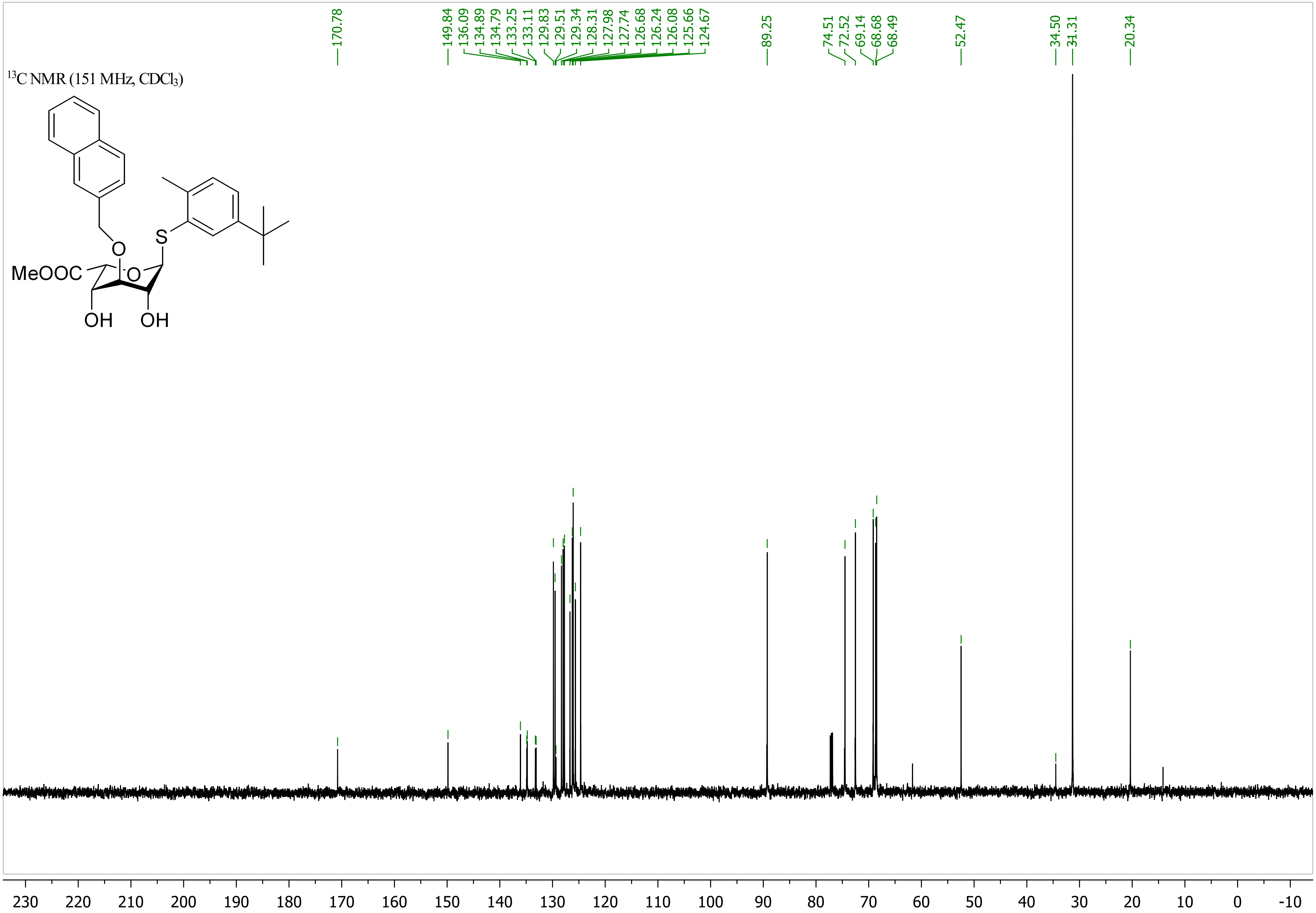
13C NMR (151 MHz, CDCl3)
170.78
149.84
136.09
134.89
134.79
133.25
133.11
129.83
129.51
129.34
128.31
127.98
127.74
126.68
126.24
126.08
125.66
124.67
89.25
74.51
72.52
69.14
68.68
68.49
52.47
34.50
31.31
20.34
MeOOC
OH
OH
O
O
S
230 220 210 200 190 180 170 160 150 140 130 120 110 100 90 80 70 60 50 40 30 20 10 0 -10

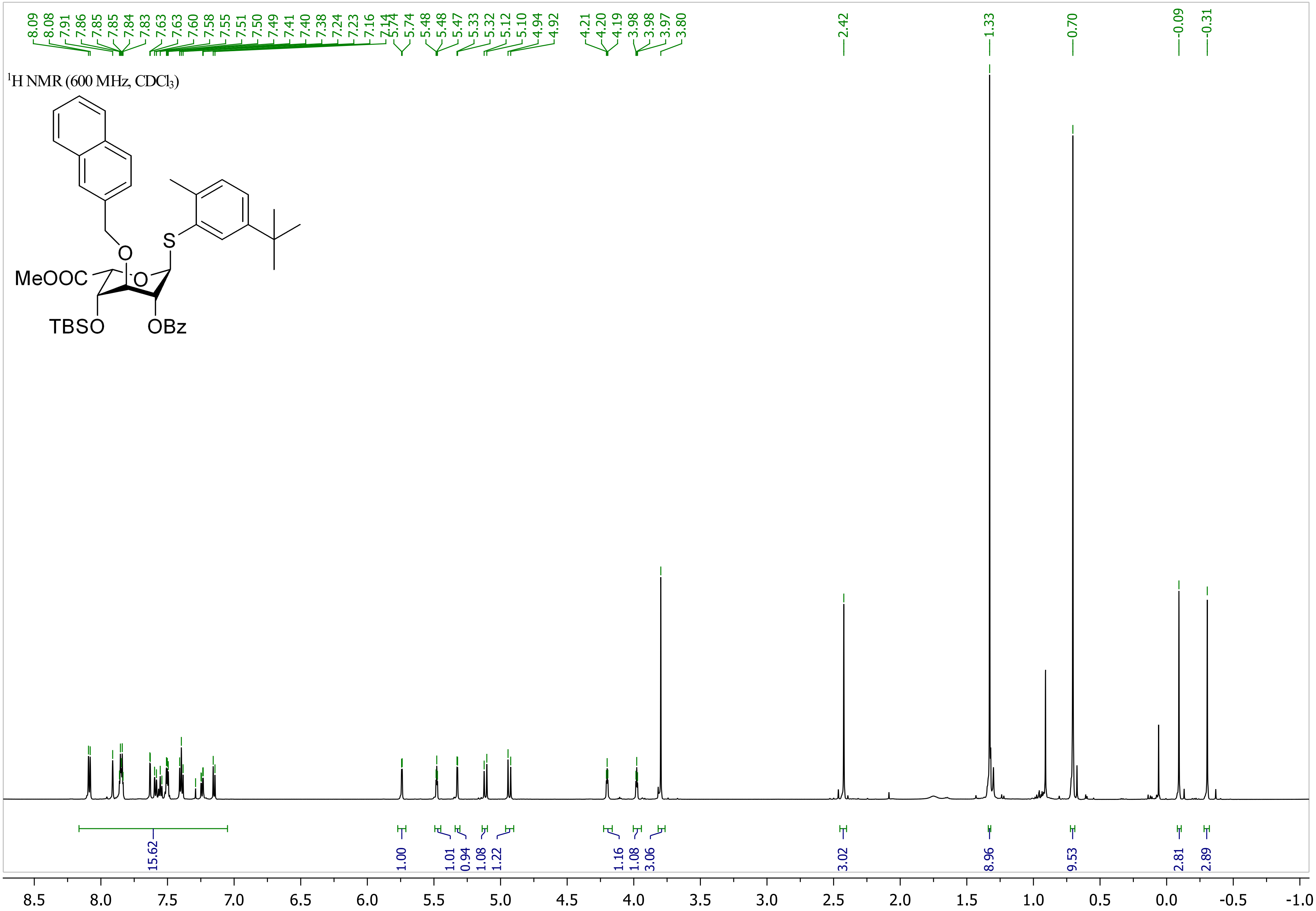

¹H NMR (600 MHz, CDCl₃)
MeOOC
TBSO
OBz
S
O
8.5 8.0 7.5 7.0 6.5 6.0 5.5 5.0 4.5 4.0 3.5 3.0 2.5 2.0 1.5 1.0 0.5 0.0 -0.5 -1.0

^{13}C NMR (151 MHz, $CDCl_3$)

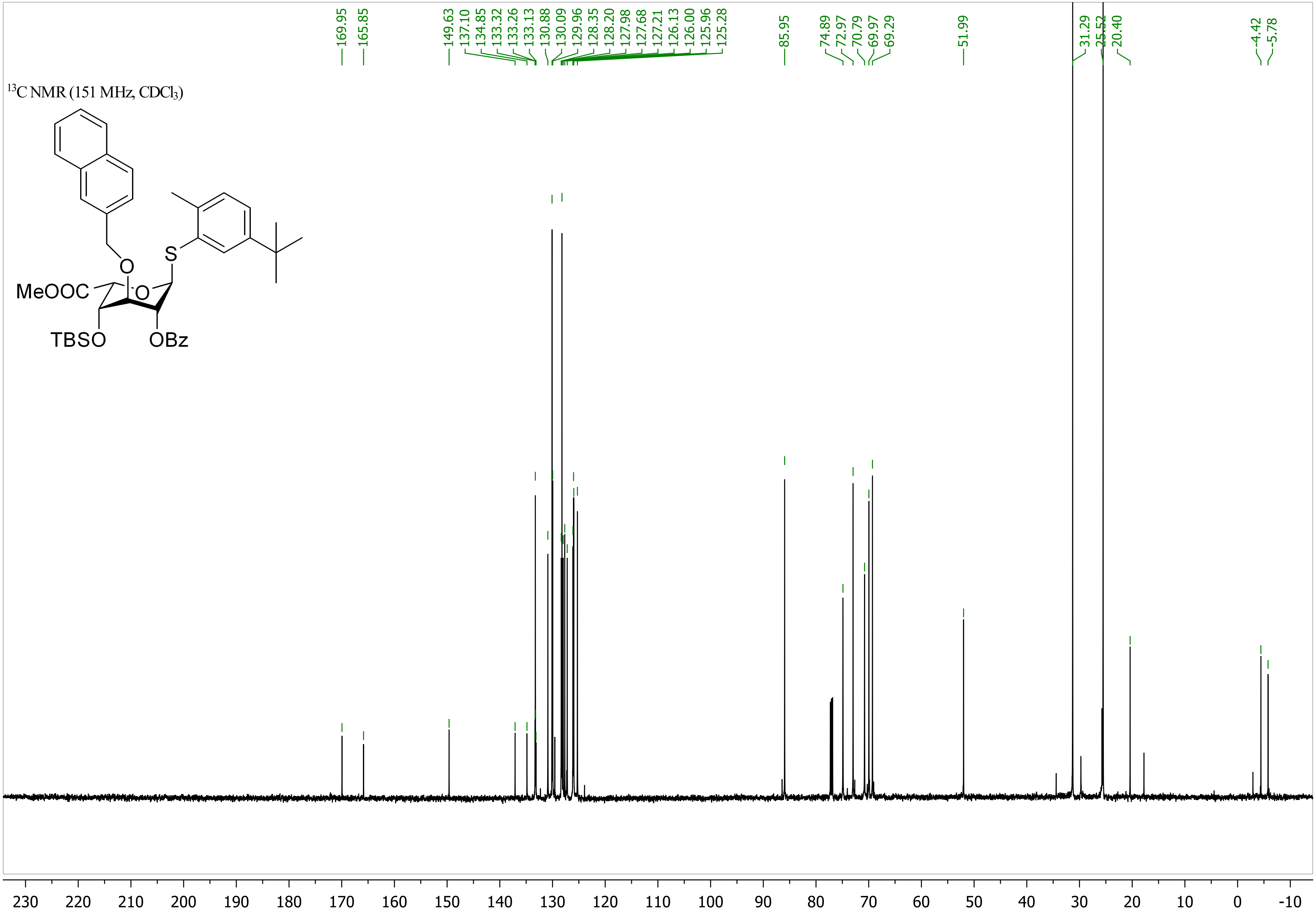

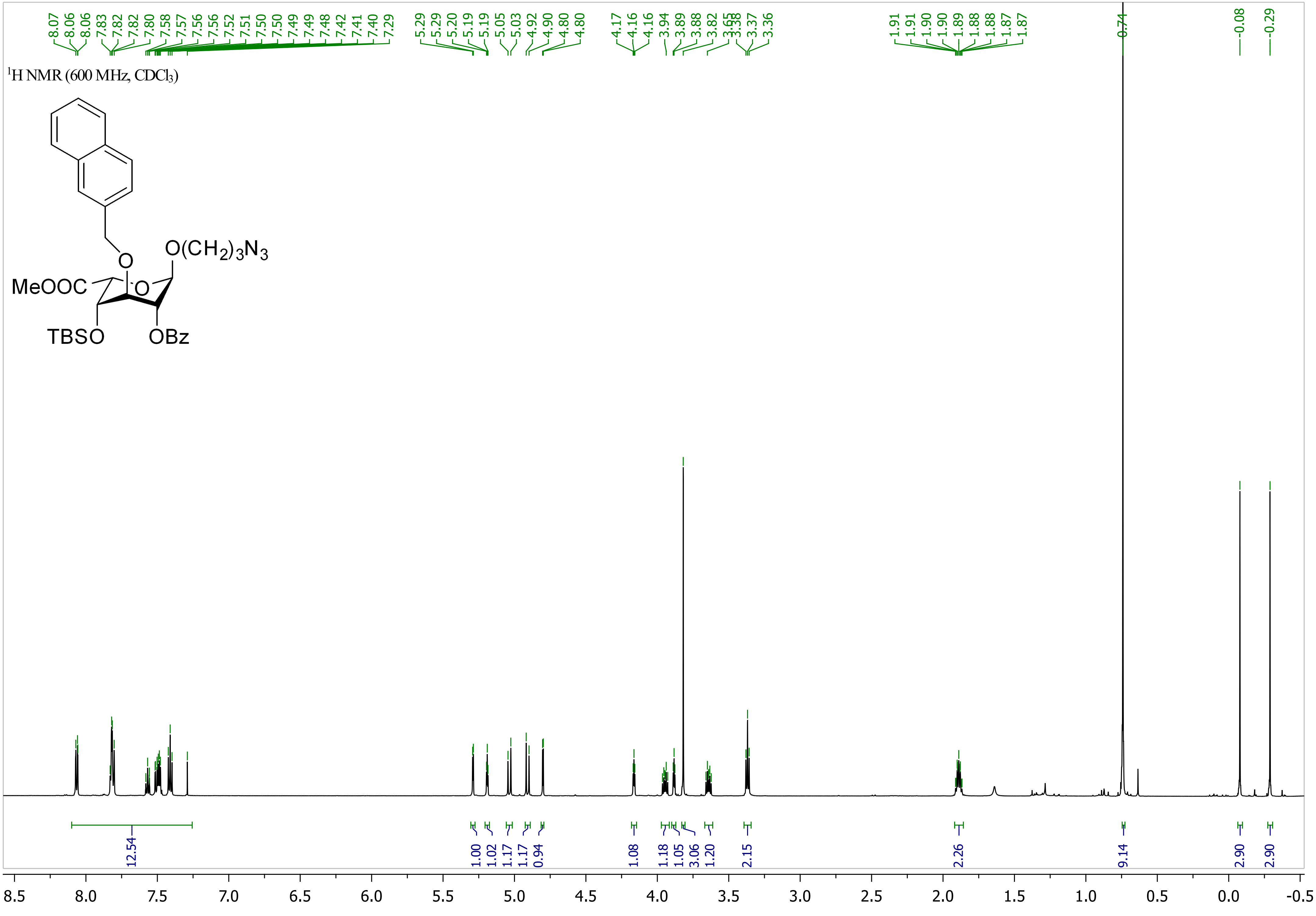

1H NMR (600 MHz, CDCl3)
O(CH2)3N3
MeOOC
O
O
TBSO
OBz
8.07 8.06 8.06 7.83 7.82 7.82 7.80 7.58 7.57 7.56 7.56 7.52 7.51 7.50 7.50 7.49 7.49 7.48 7.42 7.41 7.40 7.29
5.29 5.29 5.20 5.19 5.19 5.05 5.03 4.92 4.90 4.80 4.80
4.17 4.16 4.16 3.94 3.89 3.88 3.82 3.65 3.38 3.37 3.36
1.91 1.91 1.90 1.90 1.89 1.88 1.88 1.87 1.87
0.74
-0.08
-0.29
12.54 1.00 1.02 1.17 1.17 0.94 1.08 1.18 1.05 3.06 1.20 2.15 2.26 9.14 2.90 2.90
8.5 8.0 7.5 7.0 6.5 6.0 5.5 5.0 4.5 4.0 3.5 3.0 2.5 2.0 1.5 1.0 0.5 0.0 -0.5

^{13}C NMR (151 MHz, $CDCl_3$)

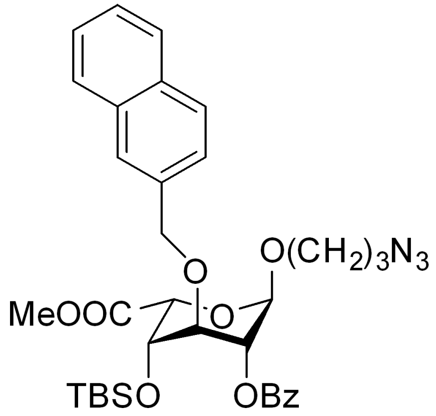

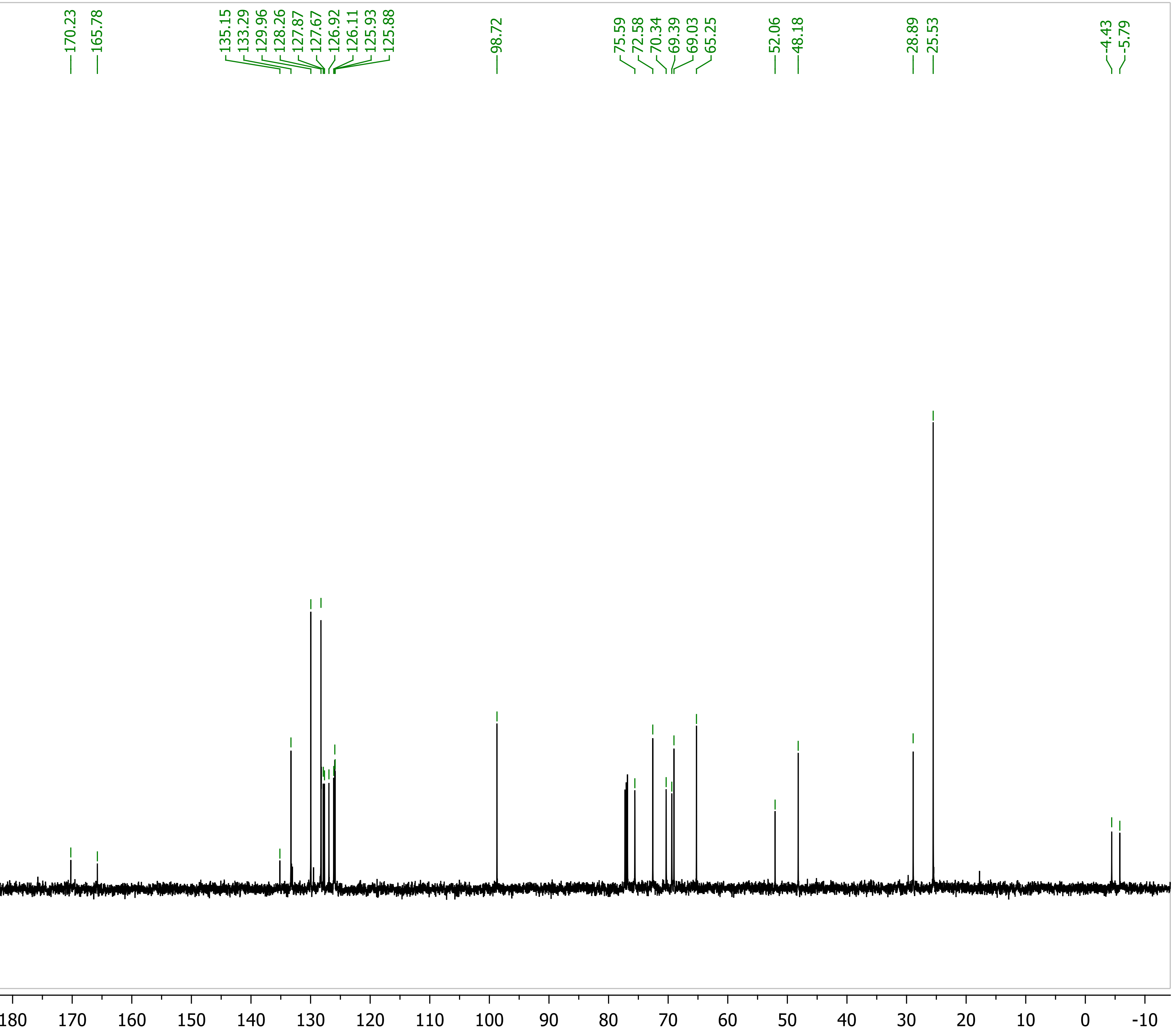

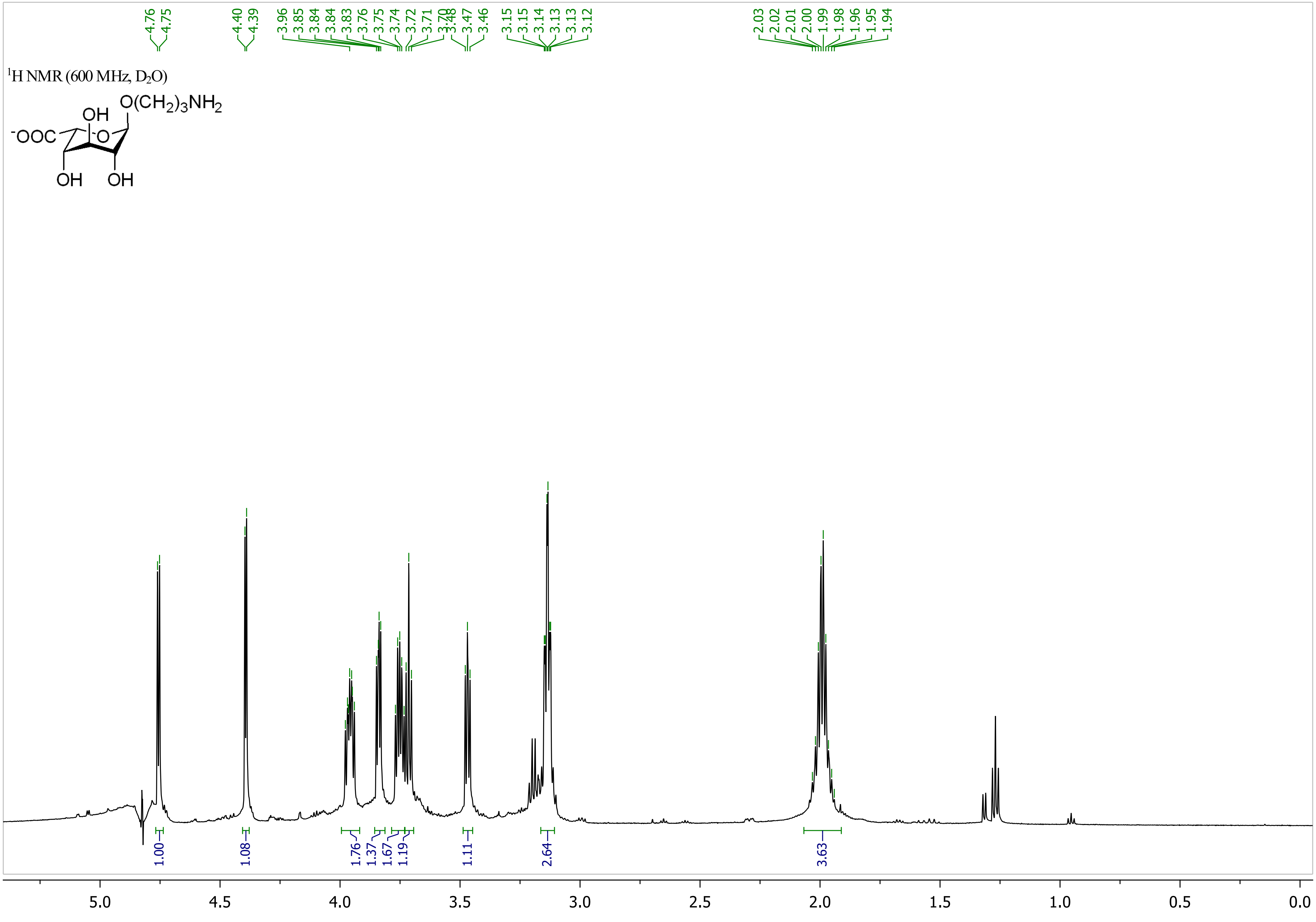
^{1}H NMR (600 MHz, D_2O)
O(CH$_2$)$_3$NH$_2$
OH
$^-$OOC
O
OH
OH
4.76 4.75 4.40 4.39 3.96 3.85 3.84 3.84 3.83 3.76 3.75 3.74 3.72 3.71 3.70 3.48 3.47 3.46 3.15 3.15 3.14 3.13 3.13 3.12 2.03 2.02 2.01 2.00 1.99 1.98 1.96 1.95 1.94
1.00 1.08 1.76 1.37 1.67 1.19 1.11 2.64 3.63
5.0 4.5 4.0 3.5 3.0 2.5 2.0 1.5 1.0 0.5 0.0

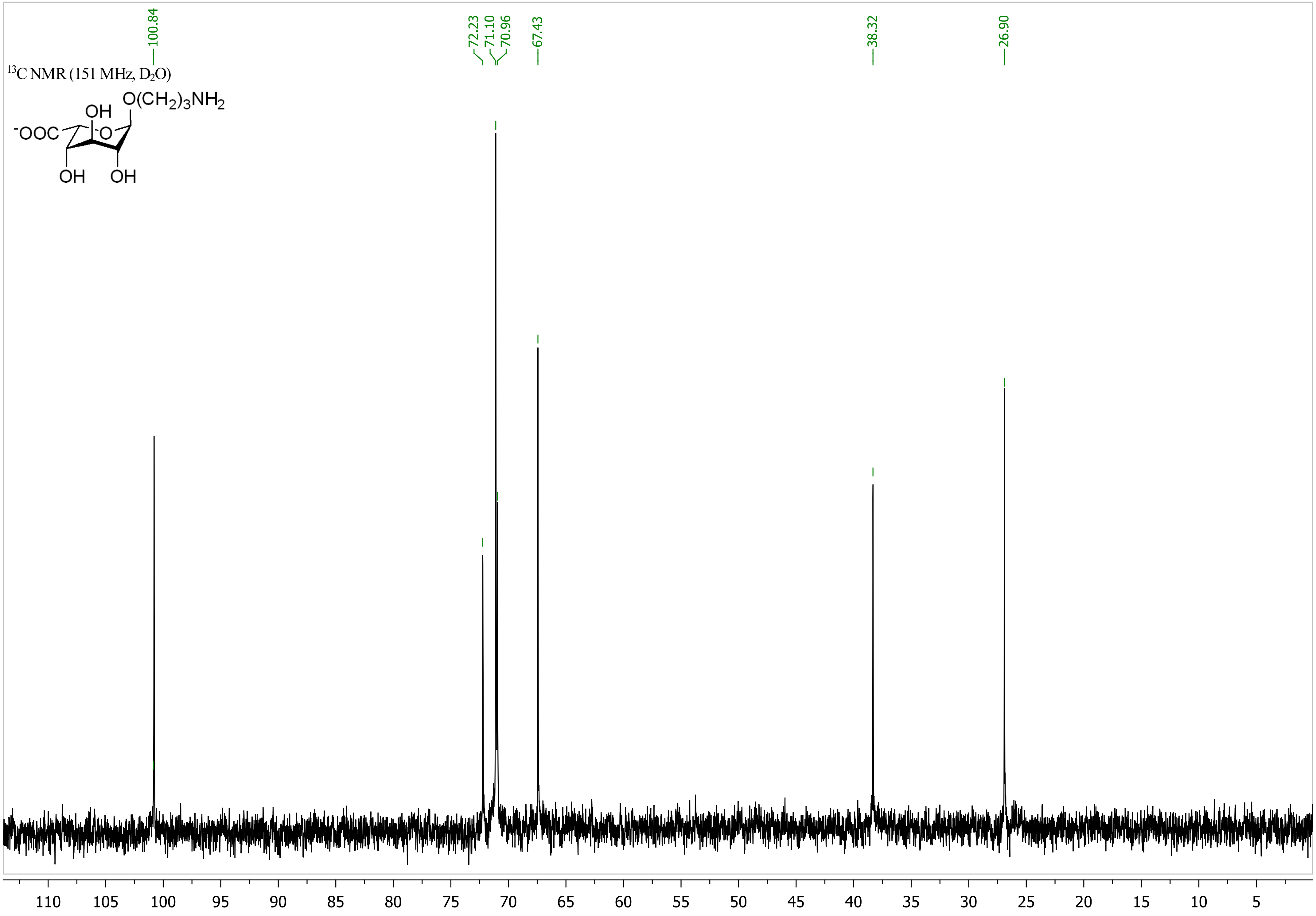

100.84
72.23
71.10
70.96
67.43
38.32
26.90
13C NMR (151 MHz, D2O)
O(CH2)3NH2
OH
-OOC
O
OH
OH
110
105
100
95
90
85
80
75
70
65
60
55
50
45
40
35
30
25
20
15
10
5

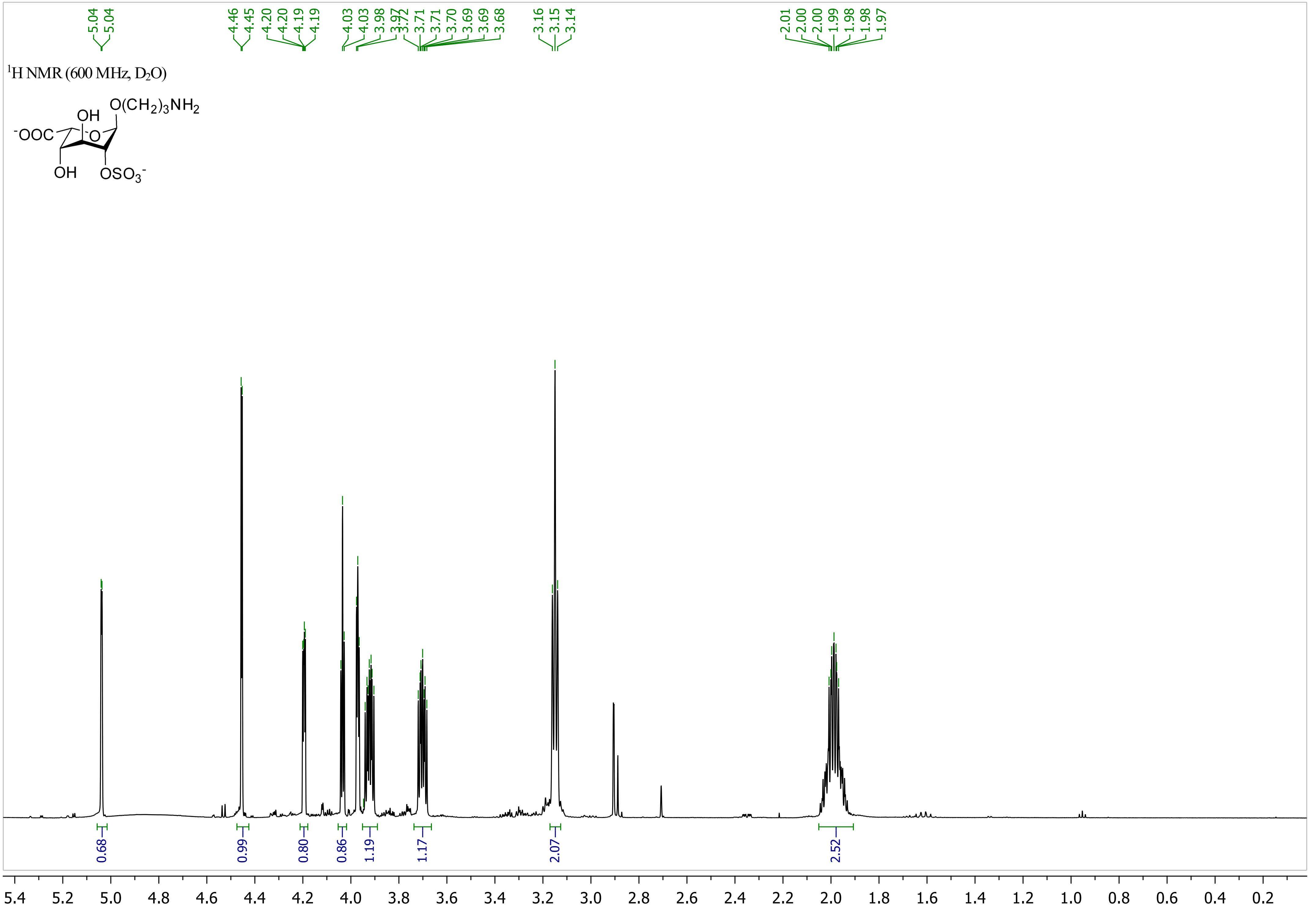

1H NMR (600 MHz, D2O)
O(CH2)3NH2
OH
-OOC
O
OH
OSO3-
5.04 5.04
4.46 4.45 4.20 4.20 4.19 4.19
4.03 4.03 3.98 3.97 3.72 3.71 3.71 3.70 3.69 3.69 3.68
3.16 3.15 3.14
2.01 2.00 2.00 1.99 1.98 1.98 1.97
0.68 0.99 0.80 0.86 1.19 1.17 2.07 2.52
5.4 5.2 5.0 4.8 4.6 4.4 4.2 4.0 3.8 3.6 3.4 3.2 3.0 2.8 2.6 2.4 2.2 2.0 1.8 1.6 1.4 1.2 1.0 0.8 0.6 0.4 0.2

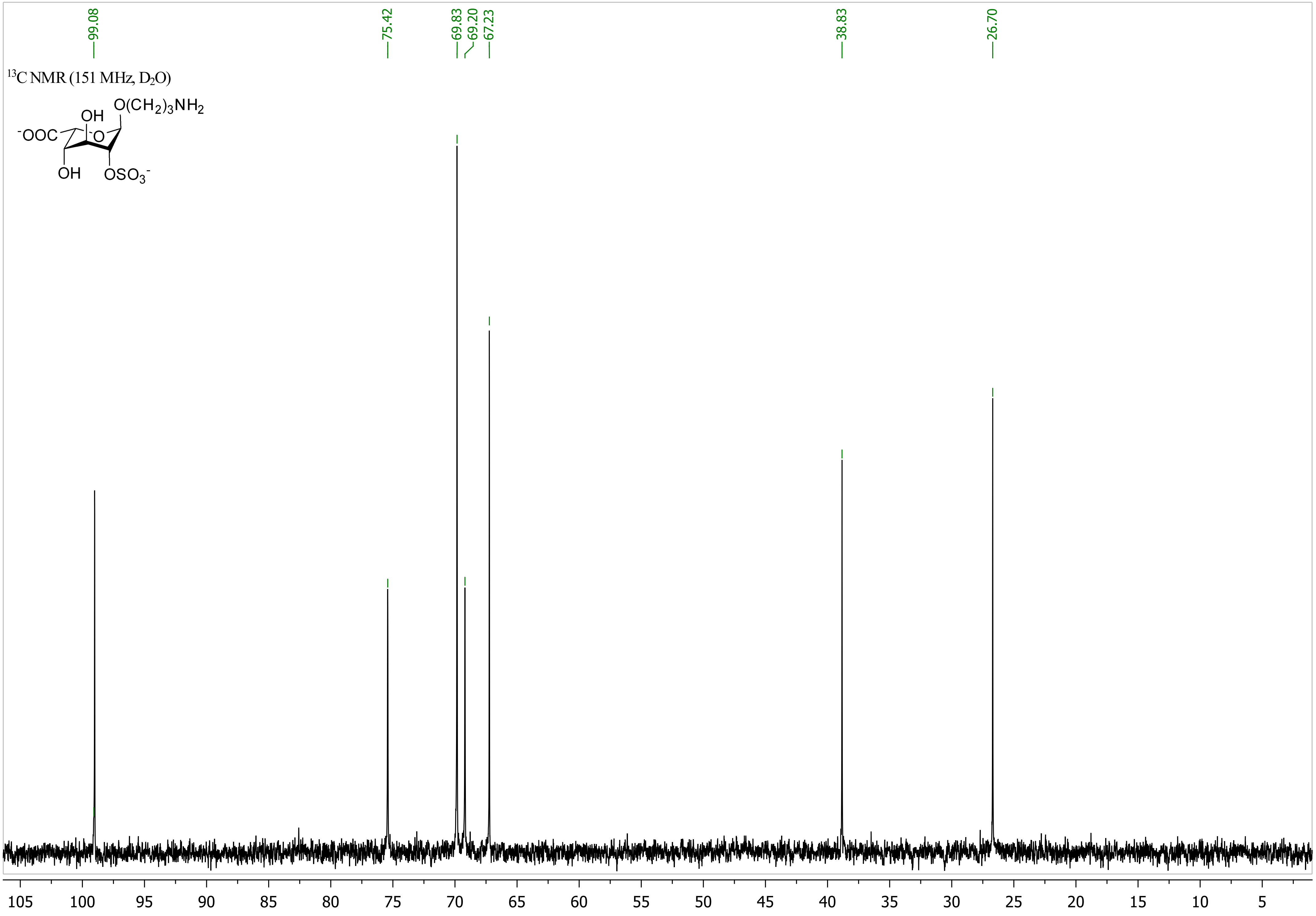

^{13}C NMR (151 MHz, D_2O)
$O(CH_2)_3NH_2$
OH
^-OOC
O
OH
OSO_3^-
99.08
75.42
69.83
69.20
67.23
38.83
26.70
105
100
95
90
85
80
75
70
65
60
55
50
45
40
35
30
25
20
15
10
5

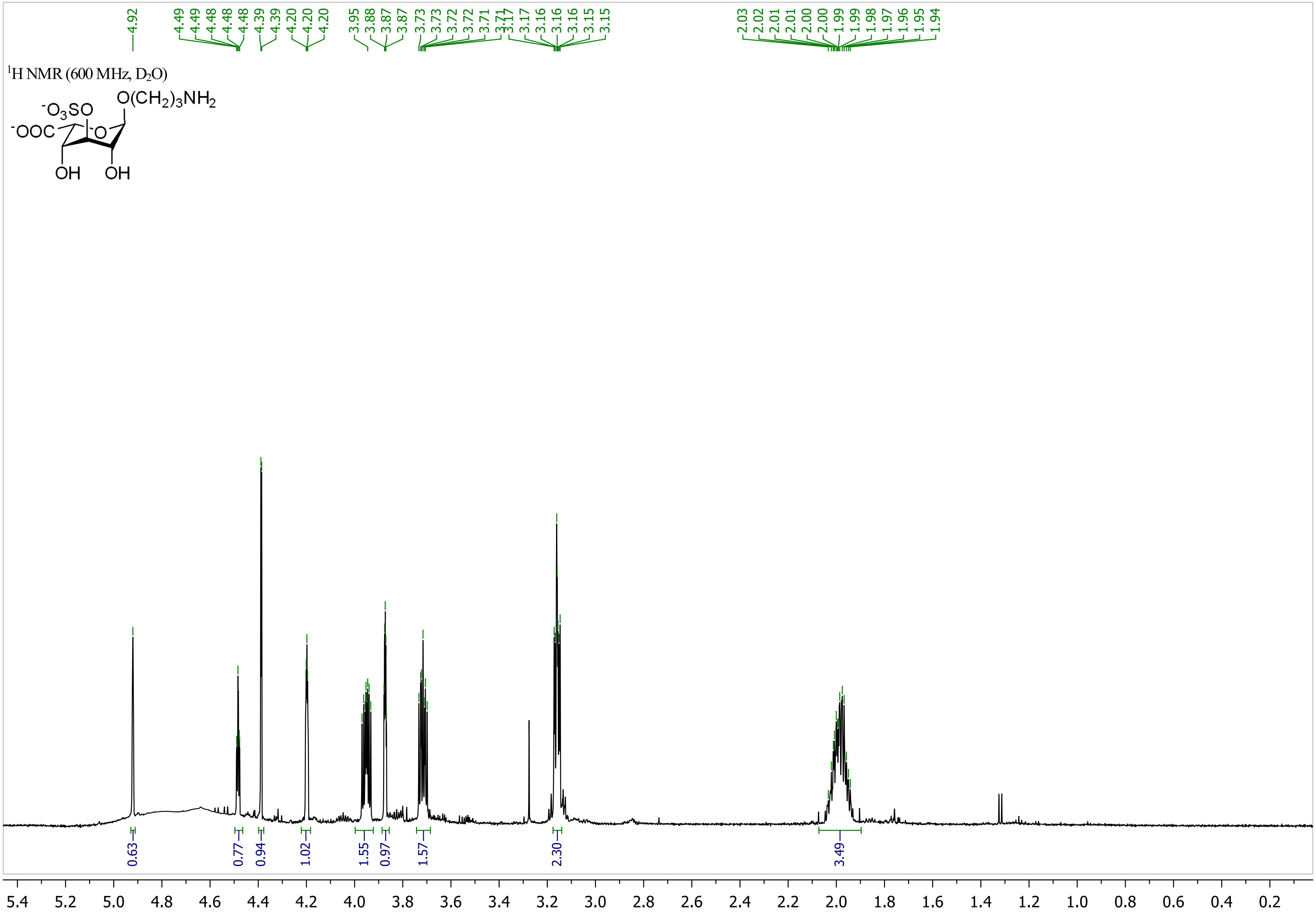

^{1}H NMR (600 MHz, D_2O)
^-O_3SO
$O(CH_2)_3NH_2$
^-OOC
O
OH
OH
4.92
4.49 4.49 4.48 4.48 4.48 4.39 4.39 4.20 4.20 4.20
3.95 3.88 3.87 3.87 3.73 3.73 3.72 3.72 3.71 3.71 3.17 3.16 3.16 3.16 3.15 3.15
2.03 2.02 2.01 2.01 2.00 2.00 1.99 1.99 1.98 1.97 1.96 1.95 1.94
0.63 0.77 0.94 1.02 1.55 0.97 1.57 2.30 3.49
5.4 5.2 5.0 4.8 4.6 4.4 4.2 4.0 3.8 3.6 3.4 3.2 3.0 2.8 2.6 2.4 2.2 2.0 1.8 1.6 1.4 1.2 1.0 0.8 0.6 0.4 0.2

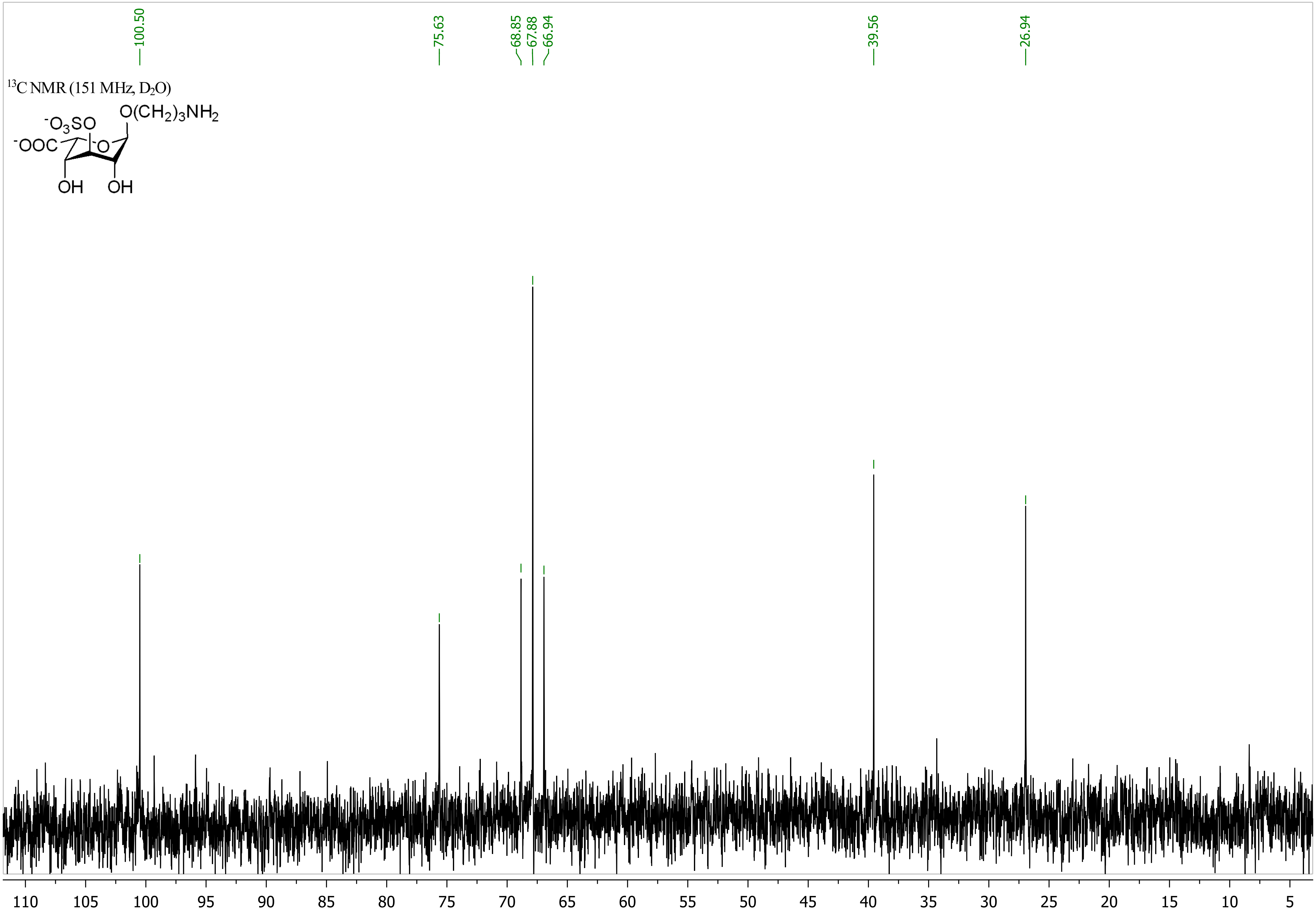
13C NMR (151 MHz, D2O)
O(CH2)3NH2
-O3SO
-OOC
O
OH
OH
100.50
75.63
68.85
67.88
66.94
39.56
26.94
110 105 100 95 90 85 80 75 70 65 60 55 50 45 40 35 30 25 20 15 10 5

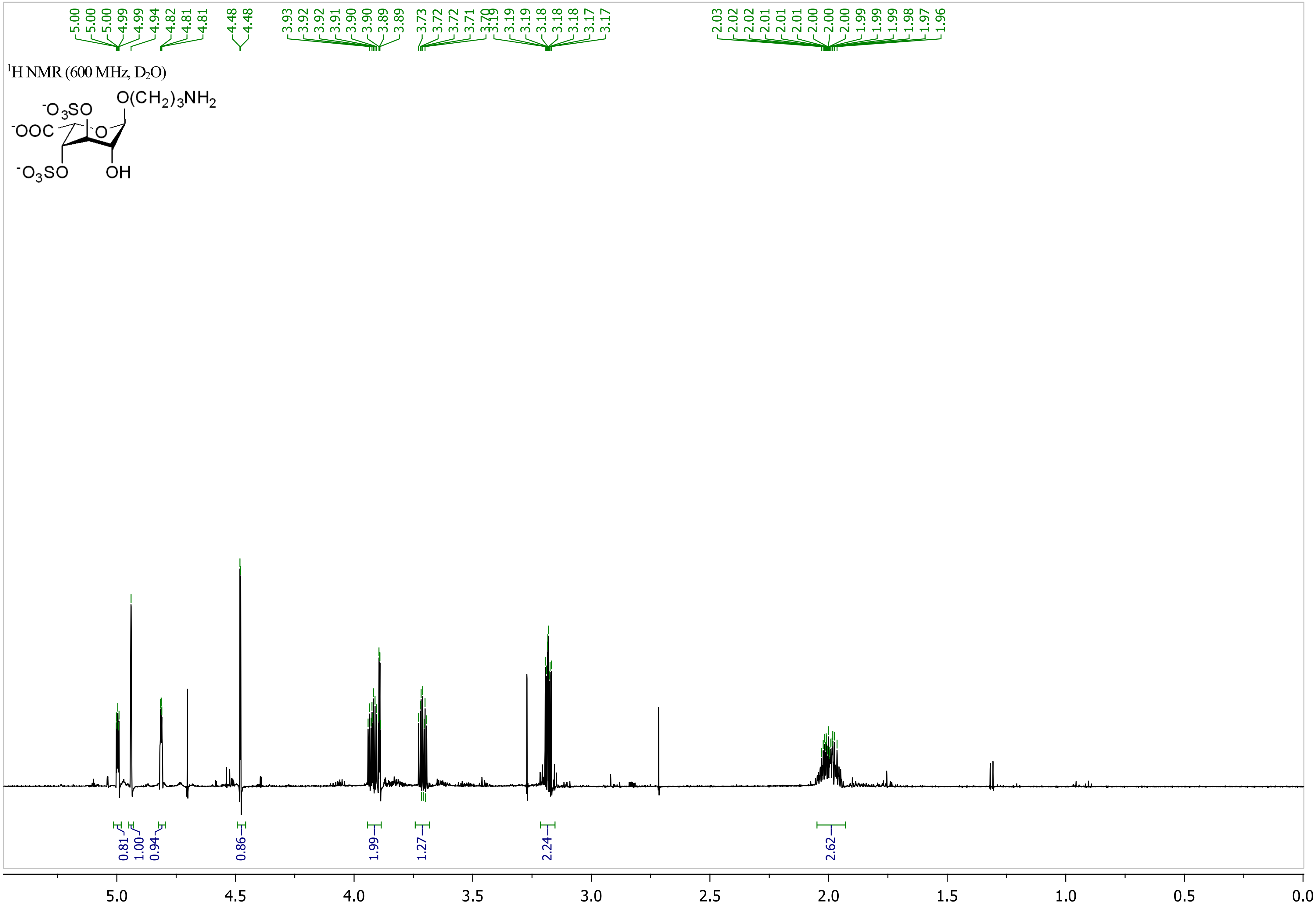

1H NMR (600 MHz, D2O)
O(CH2)3NH2
-O3SO
-OOC
O
-O3SO
OH
5.00 5.00 5.00 4.99 4.99 4.94 4.82 4.81 4.81 4.48 4.48 3.93 3.92 3.92 3.91 3.90 3.90 3.89 3.89 3.73 3.72 3.72 3.71 3.70 3.19 3.19 3.19 3.18 3.18 3.18 3.17 3.17 2.03 2.02 2.02 2.01 2.01 2.01 2.00 2.00 2.00 1.99 1.99 1.99 1.98 1.97 1.96
0.81 1.00 0.94 0.86 1.99 1.27 2.24 2.62
5.0 4.5 4.0 3.5 3.0 2.5 2.0 1.5 1.0 0.5 0.0

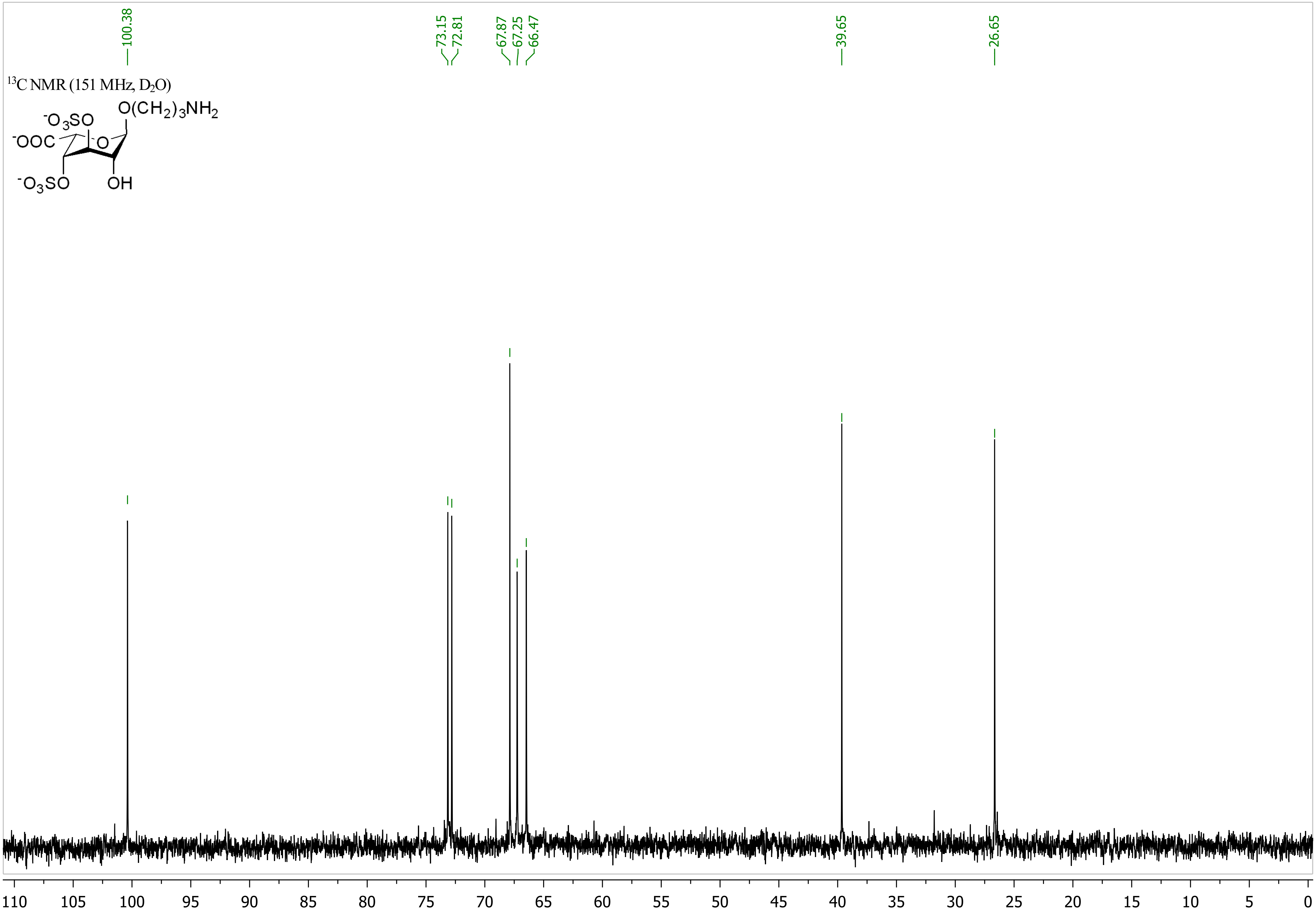

100.38
73.15
72.81
67.87
67.25
66.47
39.65
26.65
13C NMR (151 MHz, D2O)
O(CH2)3NH2
-O3SO
-OOC
O
-O3SO
OH
110
105
100
95
90
85
80
75
70
65
60
55
50
45
40
35
30
25
20
15
10
5
0

^{1}H NMR (600 MHz, D_2O)

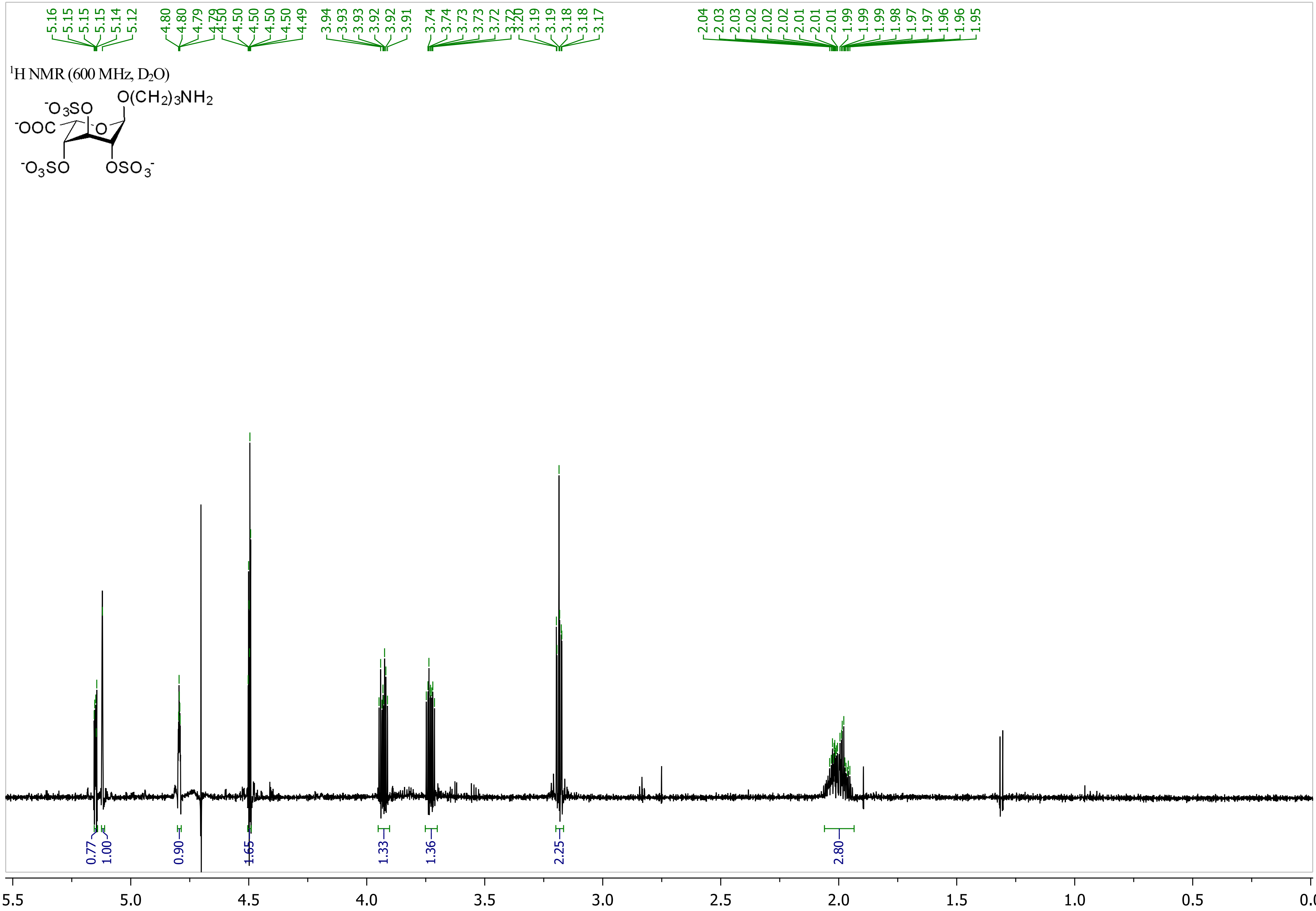

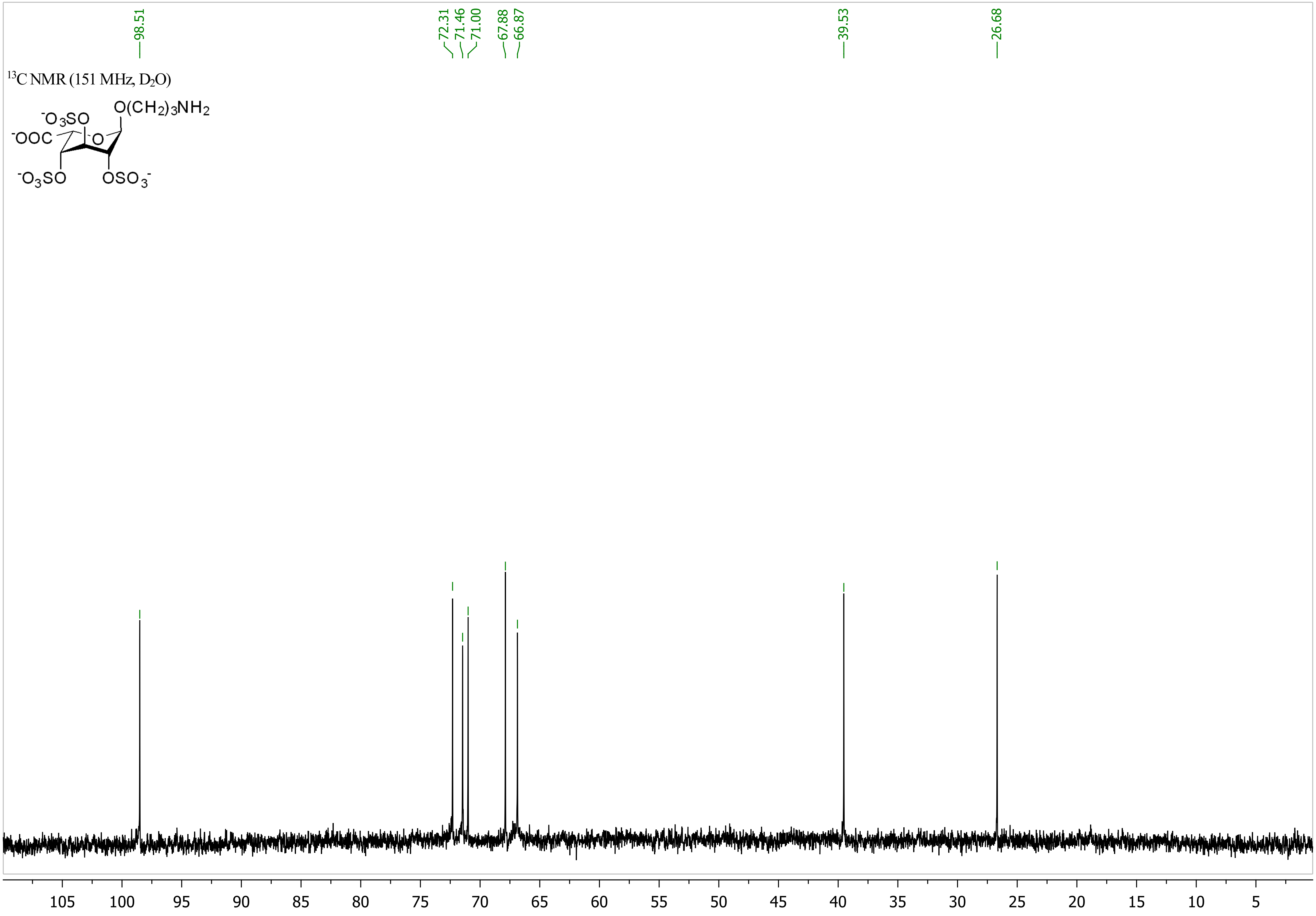

98.51
72.31
71.46
71.00
67.88
66.87
39.53
26.68
13C NMR (151 MHz, D2O)
O(CH2)3NH2
-O3SO
-OOC
O
-O3SO
OSO3-
105
100
95
90
85
80
75
70
65
60
55
50
45
40
35
30
25
20
15
10
5
